# Hydrogen releasing biomaterials for bone regeneration: mechanisms, design strategies, and applications

**DOI:** 10.1016/j.bioactmat.2026.09.002

**Published:** 2026-09-07

**Authors:** Yanshan Chen, Yuanyuan Yin, Baoyi Huang, Jingqiu Chen, Tingting Shu, Xiaohui Xu, Guangyue Li

**Affiliations:** a College of Stomatology, Chongqing Medical University, Chongqing, China; b Chongqing Key Laboratory of Oral Diseases, Chongqing, China; c Chongqing Municipal Key Laboratory of Oral Biomedical Engineering of Higher Education, Chongqing, China; d Chongqing Municipal Health Commission Key Laboratory of Oral Biomedical Engineering, Chongqing, China

## Abstract

Complex bone defects remain major clinical challenges due to their multifaceted pathological microenvironment. In recent years, hydrogen (H_2_) therapy has emerged as a rapidly developing strategy for bone repair, leveraging its redox-regulatory and other microenvironment-modulating effects. However, the rapid diffusion and short retention time of H_2_ limit its direct clinical application. The development of H_2_-releasing biomaterials partially addresses these limitations and offers a synergistic approach that integrates gas therapy with bone tissue engineering. Although recent studies on H_2_-releasing systems for bone regeneration have markedly increased, a dedicated review in this field remains absent. To address this gap, this review summarizes the pathological features of bone defect microenvironments and the multi-target biological mechanisms of H_2_ relevant to bone repair. It then discusses design objectives, optimization strategies, and classifications of H_2_-releasing biomaterials. Recent advances in their applications for bone regeneration are highlighted with an emphasis on material design and functional performance. Cross-disciplinary insights are also introduced to inspire next-generation H_2_-releasing systems for bone regeneration. Finally, the limitations and clinical translation challenges of such systems are critically discussed, and future research directions are proposed. H_2_-releasing biomaterials integrate gas therapy, materials engineering, and microenvironmental regulation, representing an emerging frontier for multi-mechanism synergistic bone regeneration.

## Highlights

• Redox-regulatory properties: H_2_ may attenuate excessive oxidative stress while exerting relatively limited effects on physiological redox processes.
• Stimuli-responsive classification: H_2_-releasing biomaterials are systematically categorized into chemically and physically responsive systems, highlighting the shift from passive release to on-demand delivery.
• Multi-mechanism synergy: Beyond single-function gas delivery, combining H_2_ therapy with bioactive ions, drugs, and physical cues opens a new paradigm for synergistic bone regeneration in complex pathological conditions.

## Graphical abstract

**Figure:**
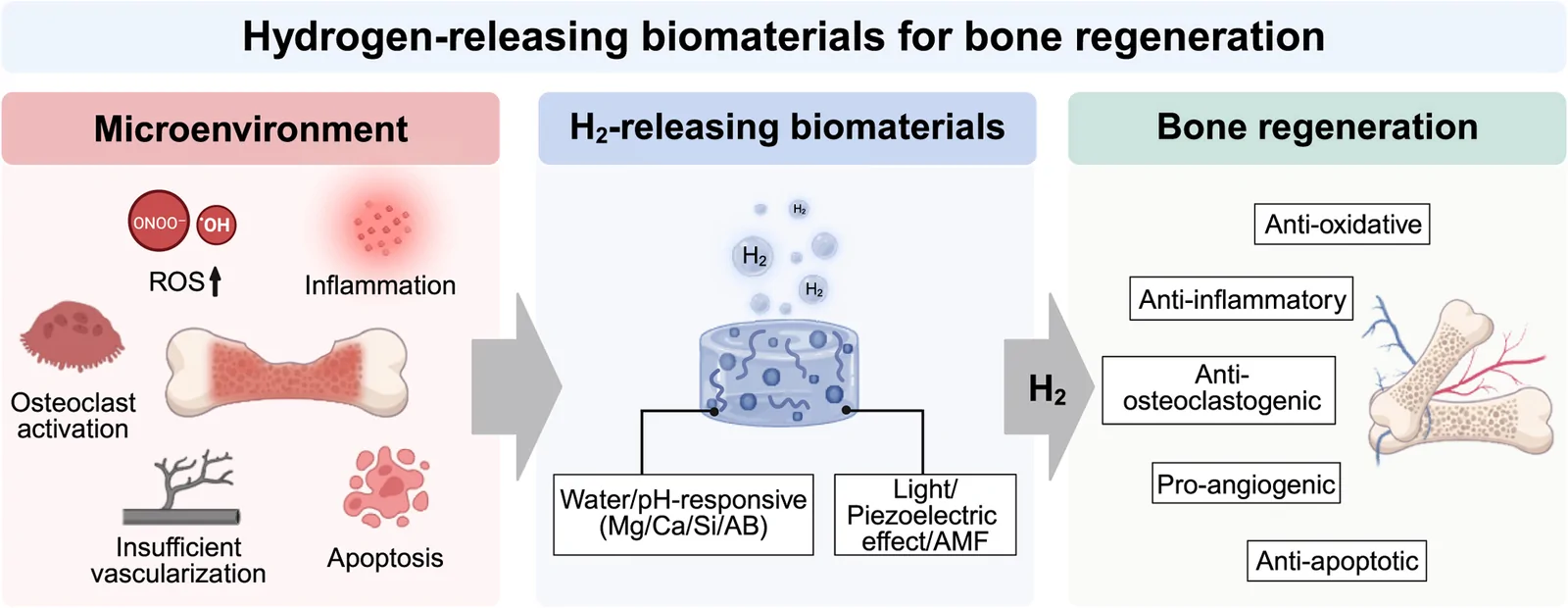

## 1 Introduction

Bone regeneration is frequently compromised under pathological conditions such as infection, diabetes, aging, and osteoporosis, posing major challenges in orthopedic and oral-maxillofacial reconstruction [[1], [2], [3], [4]]. These conditions are not driven by a single pathological process. Instead, they arise from an interactive pathological microenvironment characterized by oxidative stress imbalance [5], hyperactive immuno-inflammatory cascades [6], insufficient vascularization [7], and dysregulation of multiple osteogenic and osteoclastic signaling networks [8]. Consequently, a vicious cycle involving elevated reactive oxygen species (ROS), persistent inflammation, reduced perfusion, and impaired osteogenesis is established locally. Although conventional grafting materials such as autografts and allografts are among the most frequently chosen options clinically [9], they are often insufficient to effectively modulate such multifactorial pathological microenvironments, limiting their ability to restore local tissue homeostasis and achieve predictable functional bone regeneration [10,11].

In this context, various biomaterial based therapeutic strategies have been developed to modulate the pathological microenvironment associated with impaired bone regeneration, such as local delivery of anti-oxidative agents, growth factor-related approaches, and ion-releasing materials [[12], [13], [14], [15]]. These anti-oxidative delivery strategies typically deliver agents with anti-oxidative activity (e.g., curcumin, tocopherol, polyphenols, and ceria-based nanomaterials) locally and exert their therapeutic effects primarily through broad-spectrum ROS scavenging. However, such non-selective redox regulation may disrupt physiological ROS signaling required for osteogenesis and bone remodeling, thereby compromising the precision and coordination of tissue repair [16]. In contrast, H_2_ has been suggested to preferentially attenuate highly reactive oxidative species, particularly hydroxyl radicals (·OH) and peroxynitrite (ONOO^−^), while exerting relatively limited effects on physiological redox signaling [17,18]. This proposed redox-regulatory property may contribute to reducing oxidative damage while maintaining redox homeostasis. Beyond its anti-oxidative effects, H_2_ exerts multiple regulatory functions, including immunomodulatory, anti-apoptotic [19], anti-osteoclastogenic [20], and pro-angiogenic effects [21]. Although a direct osteoinductive effect of H_2_ has not been conclusively demonstrated, its primary therapeutic value lies in pathological microenvironment modulation, together with its excellent biosafety profile [[22], [23], [24]], making H_2_ highly complementary to conventional osteogenic components in biomaterial-based combination strategies for bone regeneration. Under physiological conditions, H_2_ is primarily generated by the gut microbiota and is thought to contribute to maintaining redox homeostasis and inflammatory balance [25,26]. However, endogenous H_2_ is generally maintained at relatively low physiological levels (0-5 μmol/L) [17,27,28].

Early investigations of H_2_ therapy for bone-related diseases primarily employed systemic delivery approaches, such as H_2_-rich water, H_2_ saline, and inhaled H_2_ [[29], [30], [31], [32], [33]]. However, systemic H_2_ delivery is constrained by rapid diffusion, low solubility, short local retention, and insufficient accumulation at defect sites, making it challenging to sustain therapeutically effective local H_2_ concentrations throughout the prolonged process of bone regeneration [34,35]. To address these limitations, recent studies have increasingly focused on localized and sustained H_2_ delivery using biomaterial-based platforms. These systems not only prolong H_2_ availability within the defect microenvironment, but also enable the co-delivery of diverse bioactive cues within biodegradable scaffolds, providing multifaceted therapeutic strategies for bone regeneration [36,37]. Based on their release mechanisms, current H_2_-releasing biomaterials for bone regeneration can be broadly categorized into chemically responsive and physically responsive systems. Chemically responsive platforms typically utilize pathological microenvironmental cues to trigger in situ H_2_ generation. In contrast, physically responsive systems rely on external stimuli, such as light, ultrasound, magnetic fields, or electrical stimulation, to enable externally triggered and tunable H_2_ release. Consequently, H_2_-releasing biomaterials are increasingly recognized as multifunctional and stimuli-responsive therapeutic platforms that integrate controlled H_2_ delivery, pathological microenvironment modulation, and coordinated support for subsequent bone regeneration processes.

**Fig. 1:**
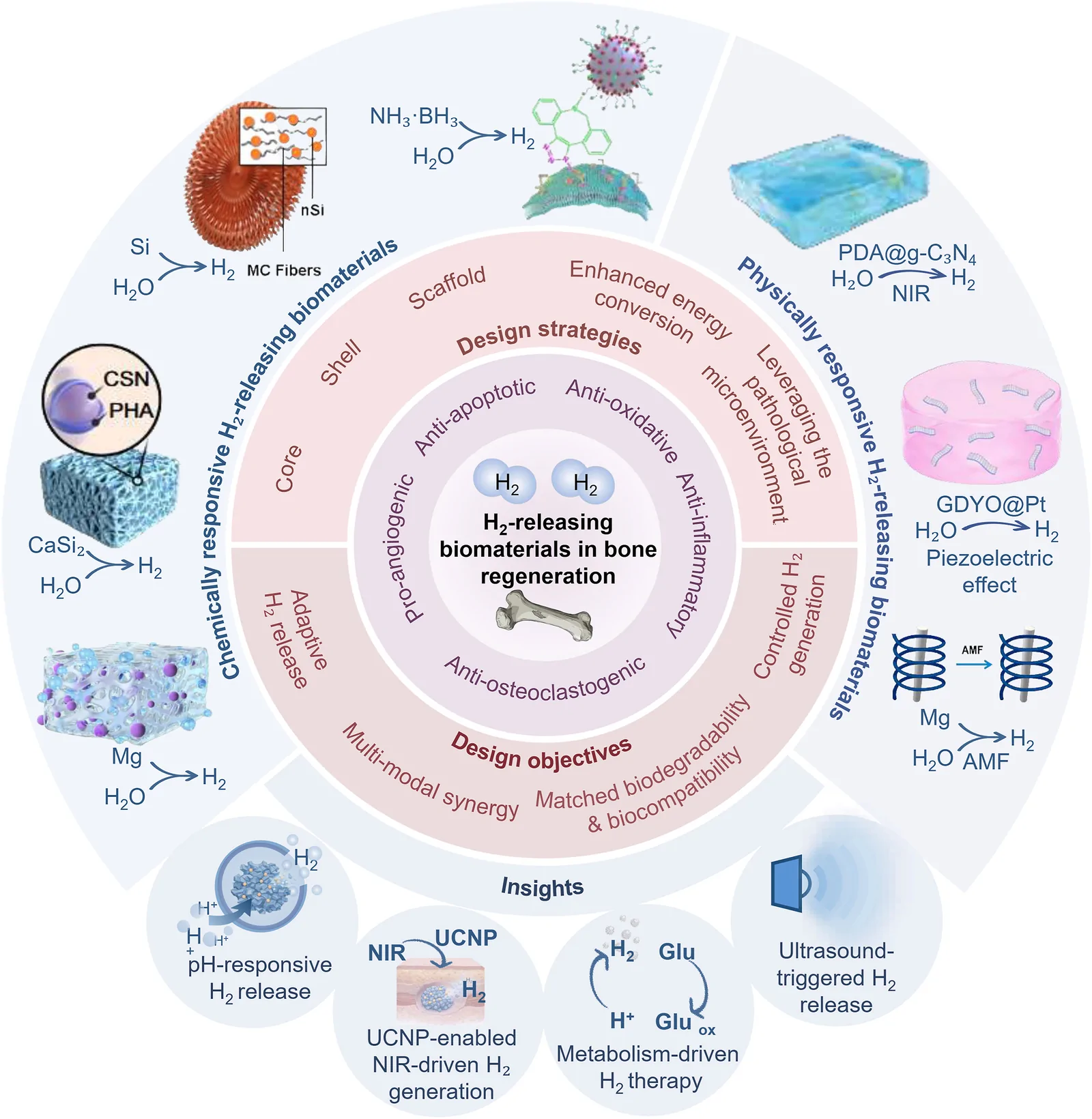
Schematic illustrating the design strategies of H_2_-releasing biomaterials in bone regeneration and their current research progress. Reproduced with permission [36,[38], [39], [40], [41], [42], [43]]. Copyright 2024, The authors; Copyright 2023, The authors; Copyright 2022, Elsevier; Copyright 2025, Elsevier; Copyright 2024, Elsevier; Copyright 2025, The authors. Copyright 2024, The authors.

Based on these advances, this review first delineates the key characteristics of the dynamic pathological microenvironment in bone defects and clarifies the biological basis underlying the broad therapeutic effects of H_2_ intervention. It then outlines the design objectives and strategies for H_2_-releasing biomaterials, followed by a systematic classification into chemically responsive and physically responsive systems. Recent progress over the past five years is highlighted, with an emphasis on design strategies and functional performance. Subsequently, drawing inspiration from advanced design concepts in other biomedical fields, this review discusses how cross-disciplinary insights can inform the development of H_2_-releasing systems for bone regeneration. Finally, we address the current limitations and challenges facing the field, along with future directions for improvement, aiming to promote a multi-mechanism synergistic paradigm for bone regeneration (Fig. 1).

## 2 Pathological microenvironment of bone defects

Bone defect repair is a highly dynamic process involving synergistic microenvironmental reconstruction, encompassing multiple interconnected biological stages such as redox homeostasis regulation, immune cell infiltration and phenotype switching, osteoclast-osteoblast coupling, angiogenesis, stem cell recruitment and differentiation, as well as matrix degradation and remodeling [5,44,45]. Normal bone healing follows a sequential progression through inflammatory, reparative, and remodeling phases [46]. The early inflammatory phase, dominated by neutrophils and macrophages, involves the release of ROS and pro-inflammatory factors to clear necrotic tissue and stimulate angiogenic factors [47]. Subsequently, inflammation shifts toward a pro-repair phenotype [[48], [49], [50]], accompanied by microvascular reconstruction and osteogenic differentiation of mesenchymal stem cells (MSCs) [51,52], ultimately leading to matrix mineralization. Any temporal misalignment or dysregulated intensity during these stages, such as excessive inflammation or sustained high ROS levels, can lead to delayed bone healing, incomplete union, or nonunion [53,54].

Oxidative stress plays a central role in the pathological microenvironment of bone defects. On one hand, transient and controlled ROS act as signaling molecules to promote angiogenesis and osteogenic signaling, for instance, through amplification of the HIF-1α/VEGF pathway under hypoxia-reoxygenation conditions [55,56]. On the other hand, excessive ROS, particularly strong oxidants such as ·OH and ONOO^−^, can damage mitochondria, induce DNA and lipid peroxidation, and cause protein denaturation. This triggers apoptosis or cellular senescence, inhibits the osteogenic differentiation of MSCs, and enhances osteoclast activity [57,58], thereby exacerbating bone resorption and leading to repair failure. Consequently, maintaining an appropriate redox balance is crucial for the osteogenic microenvironment [59].

Imbalance in the immune microenvironment is another key factor contributing to impaired bone regeneration. Although an initial transient inflammatory phase is necessary for bone healing [60], chronic inflammation or a failure to switch from a pro-inflammatory to a reparative phenotype leads to the sustained secretion of inflammatory cytokines (TNF-α, IL-1β, and IL-6). This pro-inflammatory microenvironment sustains M1-like macrophages, inhibits MSC osteogenesis, and drives osteoclastogenesis via the RANKL pathway, ultimately causing a pathological state dominated by bone resorption [61,62]. Therefore, timely regulation of both immune phenotypic switching (specifically from M1 to M2) and the suppression of excessive inflammatory responses represents a pivotal strategy for promoting bone regeneration.

Aberrant osteoclast differentiation is another critical mechanism contributing to failed bone regeneration within the pathological microenvironment characterized by sustained inflammation and oxidative stress. Excessive ROS and pro-inflammatory factors such as TNF-α, IL-1β, and IL-6 upregulate the RANKL/OPG ratio, persistently activating the RANK–NF-κB–NFATc1 signaling axis. This drives excessive differentiation of monocytes/macrophages into osteoclasts and enhances their bone-resorptive activity [63,64]. Furthermore, ROS serve as critical mediators that amplify RANKL signaling, maintaining osteoclast activity through disruption of mitochondrial homeostasis [65]. Concurrently, chronic inflammation further suppresses osteoblast maturation and mineralization [66], ultimately disrupting the osteoblast-osteoclast coupling balance and establishing a pathological state dominated by bone resorption. Therefore, the synergistic inhibition of inflammation and oxidative stress, along with restoring osteoblast-osteoclast balance, is a crucial strategy in the design of bone repair materials to promote bone regeneration.

Angiogenesis and local blood perfusion play essential roles in bone regeneration. Newly formed blood vessels not only supply oxygen and nutrients to osteoblasts but also establish a vascular-bone stem cell niche, wherein signaling factors secreted by endothelial cells, such as VEGF and angiopoietins, directly regulate the proliferation and osteogenic differentiation of MSCs [67]. Insufficient vascular supply or impaired revascularization restricts osteoblast metabolic activity, delays matrix deposition, and increases the risk of local tissue necrosis [68]. In contrast, timely and functional revascularization significantly promotes bone healing [69]. Therefore, in the design of bone repair materials, it is essential to actively promote physiological angiogenesis while avoiding pathological vascular formation, thereby optimizing the regenerative microenvironment and promoting bone regeneration.

At the level of cell fate regulation, mitochondrial function, endoplasmic reticulum homeostasis, and cellular signaling pathways (e.g., NF-κB, MAPK, PI3K/AKT, Wnt/β-catenin, and Notch) collectively determine the survival and differentiation trajectory of MSCs and osteoprogenitor cells [[70], [71], [72]]. Chronic inflammation and excessive accumulation of ROS mutually promote each other, perpetuating the amplification of inflammatory responses and thereby inhibiting Wnt/β-catenin/Runx2-mediated osteogenic transcription and inducing caspase-dependent apoptosis in osteoblasts [63,73]. Therefore, preserving cellular viability and supporting osteogenic-angiogenic coupling are essential within the pathological context of bone defects. This involves regulating oxidative stress, maintaining mitochondrial and endoplasmic reticulum homeostasis, activating cell-survival signals such as PI3K/AKT, and inhibiting caspase-dependent apoptosis.

In summary, the pathological microenvironment of bone defects constitutes a complex network formed by the interplay of multiple factors, including redox imbalance, chronic inflammation, abnormal osteoclast differentiation, impaired vascular remodeling, and aberrant activation of apoptotic signaling. In this context, H_2_-releasing biomaterials have shown potential to facilitate bone regeneration due to their ability to perform multi-target intervention within the pathological microenvironment.

## 3 Biological mechanisms of H_2_

**Fig. 2:**
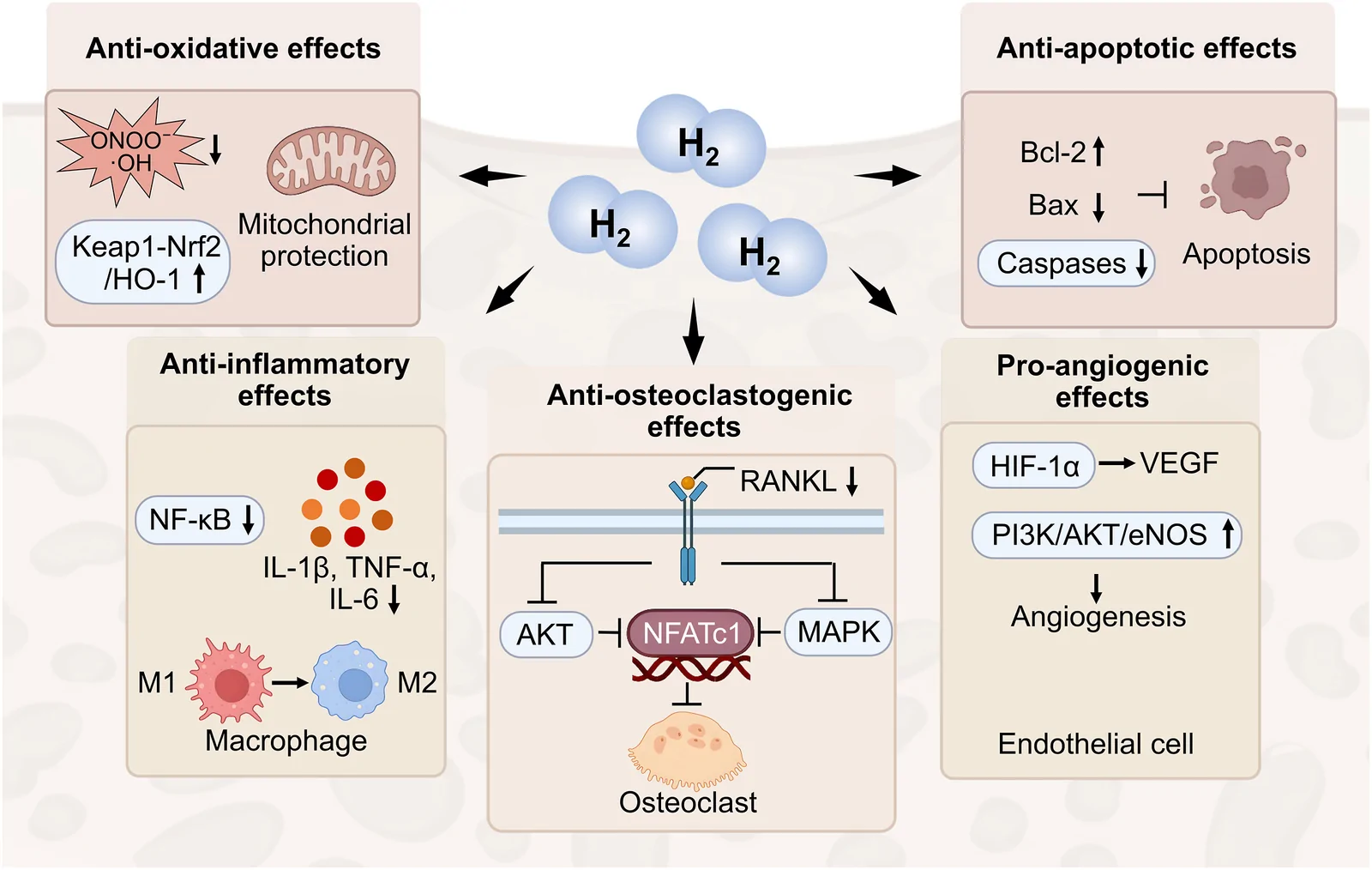
Schematic illustration of the proposed multifaceted biological mechanisms associated with H_2_-mediated bone regeneration. Created with BioRender.com.

With ongoing research, H_2_ has attracted increasing attention for its multifaceted biological activities, which mostly arise from its redox-regulatory mechanism and extend to various cellular processes. Studies have shown that this redox-regulating property has been able to preserve mitochondrial function, maintain intracellular redox homeostasis, and subsequently regulate multiple downstream biological processes [74,75], including immunomodulation, inhibition of osteoclast differentiation, promotion of angiogenesis, and anti-apoptotic activity. This multidimensional mode of action endows H_2_ with inherent advantages in reconstructing the complex microenvironment of bone defects, thereby providing a solid mechanistic foundation for the design of H_2_-releasing biomaterials. Therefore, this section aims to systematically elucidate the multifaceted biological effects of H_2_, thus facilitating the translation from understanding of molecular mechanisms to the design of biomaterials for bone regeneration (Fig. 2).

### 3.1 Anti-oxidative effects

Since Ikuroh Ohsawa and colleagues first proposed the selective anti-oxidative capacity of H_2_ in 2007 [17], numerous studies have provided evidence supporting this phenomenon. H_2_ has been reported to preferentially attenuate oxidative damage associated with highly cytotoxic ROS such as ·OH and ONOO^−^, while exerting relatively limited effects on certain physiological ROS involved in signal transduction, including H_2_O_2_ and superoxide anion (O_2_^−^) [76,77]. Unlike conventional broad-spectrum anti-oxidative agents, H_2_ has been suggested to attenuate oxidative damage while preserving redox-dependent physiological signaling pathways [78]. However, the molecular basis underlying this selective anti-oxidative phenomenon remains incompletely understood. No definitive molecular target or receptor for H_2_ has yet been identified. Moreover, increasing evidence suggests that modulation of endogenous anti-oxidative systems may contribute substantially to the biological actions of H_2_. For example, H_2_ has been reported to activate the Keap1-Nrf2 pathway and upregulate enzymes with anti-oxidative activity, such as heme oxygenase-1 (HO-1) and superoxide dismutase [76,78,79]. Given that acute oxidative burst was a common feature during early tissue repair, these anti-oxidative effects hold promise for alleviating oxidative damage and preserving the function of osteoblasts and endothelial cells in the initial stage of bone defects, thereby creating a favorable microenvironment for subsequent regeneration.

### 3.2 Anti-inflammatory effects

H_2_ was reported to mediate anti-inflammatory effects through a multi-cellular network at the immune-regulatory level. It was shown to suppress ROS-dependent activation of NF-κB and the NLRP3 inflammasome, lowering pro-inflammatory cytokines (IL-1β, TNF-α, IL-6) and promoting macrophage polarization from M1 to the reparative M2 phenotype [77,80,81]. In T cells (Th17, Th1, CD8^+^), H_2_ was found to enhance mitochondrial oxidative phosphorylation, driving a controlled increase in mitochondrial ROS that remained non-cytotoxic. This metabolic shift inhibited the production of IL-26 and IFN-γ without significantly affecting Treg function [82]. H_2_ was further shown to stabilize mitochondrial integrity by modulating mitochondrial dynamics, specifically attenuating stress-induced fragmentation through downregulation of Drp1 and upregulation of MFN1/2 or OPA1. This restoration of network architecture limited mtROS overproduction and thereby reduced inflammation-linked apoptosis [80,83]. Together, these mechanisms revealed a synergistic network where H_2_ modified immune phenotypes, reprogrammed metabolism, and improved mitochondrial performance. This integrated action broke the ROS-inflammation cycle, supported cellular homeostasis, and created a pro-regenerative tissue microenvironment.

### 3.3 Anti-osteoclastogenic effects

In bone metabolism, H_2_ was reported to inhibit osteoclast differentiation primarily through modulation of the RANKL signaling cascade. Mechanistically, H_2_ was shown to scavenge intracellular ROS to suppress RANKL-induced osteoclastogenesis, attenuating activation of the NF-κB, MAPK, and AKT pathways and downregulating the master transcription factor NFATc1 [84]. RNA sequencing of bone marrow-derived mononuclear cells treated with H_2_ found that the differentially expressed genes were involved in osteoclast differentiation cascades [20]. The anti-osteoclastogenic effect of H_2_ was confirmed in bone marrow mononuclear cells in a concentration-dependent manner [85]. In vivo study further validated this effect, showing that H_2_ selectively suppressed pathological osteoclast activation and bone resorption without significantly impairing osteoblast function in an osteoporosis Zebrafish model [86]. Collectively, these findings suggest that H_2_ may regulate osteoclastogenesis to suppress bone resorption, highlighting its potential role in maintaining bone homeostasis.

### 3.4 Pro-angiogenic effects

The role of H_2_ in promoting angiogenesis has been increasingly recognized. Studies demonstrated that H_2_ stabilized and upregulated HIF-1α, thereby activating the expression of its key downstream pro-angiogenic factor VEGF, which effectively enhanced endothelial cell migration, proliferation, and tube formation [87,88]. H_2_ was also found to restore the proliferative, migratory, and tubular formation capacities of endothelial progenitor cells by activating the PI3K/AKT/eNOS pathway [89]. Bone repair is critically dependent on neovascularization, which not only supplies nutrients but also orchestrates the osteogenic process through “vascular-osteogenic coupling”; consequently, insufficient vascularization often leads to repair failure [90]. The pro-angiogenic effect of H_2_ is therefore significant, as it contributes to improving blood flow in the acute phase and providing a favorable long-term bone metabolic microenvironment. However, studies systematically elucidating the molecular mechanisms of H_2_-mediated angiogenesis remained relatively limited.

### 3.5 Anti-apoptotic effects

In terms of cell survival and anti-apoptosis, H_2_ has been reported to exert synergistic effects through multiple pathways. Its core mechanism has been proposed to involve the preferential scavenging of highly toxic ROS, such as ·OH and ONOO^−^, which may contribute to maintaining mitochondrial membrane potential, preventing the release of cytochrome *c*, and thereby blocking the initiation of the mitochondrial apoptotic pathway [91,92]. Based on this, H_2_ was further found to modulate the expression of key apoptosis-related factors such as Bcl-2 and Bax, to suppress multiple caspase cascades including Caspase-3, Caspase-8, and Caspase-9, and to activate cell survival signaling pathways such as PI3K/AKT [23,[93], [94], [95]]. Moreover, H_2_ was demonstrated to alleviate endoplasmic reticulum stress and to suppress its mediated CHOP-dependent apoptosis [96,97]. The integrated regulation of oxidative stress, mitochondrial function, and apoptotic signaling networks collectively constituted the molecular mechanistic basis for H_2_-mediated cytoprotection.

In summary, the biological effects of H_2_ are mediated not by a single pathway but by an interconnected regulatory network. Current evidence suggests that these effects are closely linked to the ability of H_2_ to modulate cellular redox homeostasis and downstream signaling pathways, contributing to anti-inflammatory activity, inhibition of osteoclast differentiation, promotion of angiogenesis, and enhanced cell survival. Multiple signaling pathways, including Nrf2, NF-κB, MAPK, PI3K/AKT, and HIF-1α, have been implicated in H_2_-mediated biological responses, highlighting the pleiotropic nature of its biological functions. Despite substantial progress, the precise molecular basis underlying H_2_ sensing and the upstream events linking H_2_ to cellular redox regulation remain to be elucidated. Consequently, it remains difficult to determine whether the reported anti-inflammatory, anti-osteoclastogenic, pro-angiogenic, and cytoprotective effects arise from direct modulation of specific signaling pathways by H_2_ or represent secondary consequences of its regulation of cellular redox homeostasis [79,98,99]. Furthermore, mechanistic studies of H_2_ in the context of bone repair remain relatively limited. A deeper understanding of how H_2_ is sensed and translated into biological responses within the bone-defect microenvironment may further facilitate the rational design of H_2_-releasing biomaterials.

## 4 Design objectives, strategies, and classification of H_2_-releasing biomaterials

### 4.1 Design objectives

**Fig. 3:**
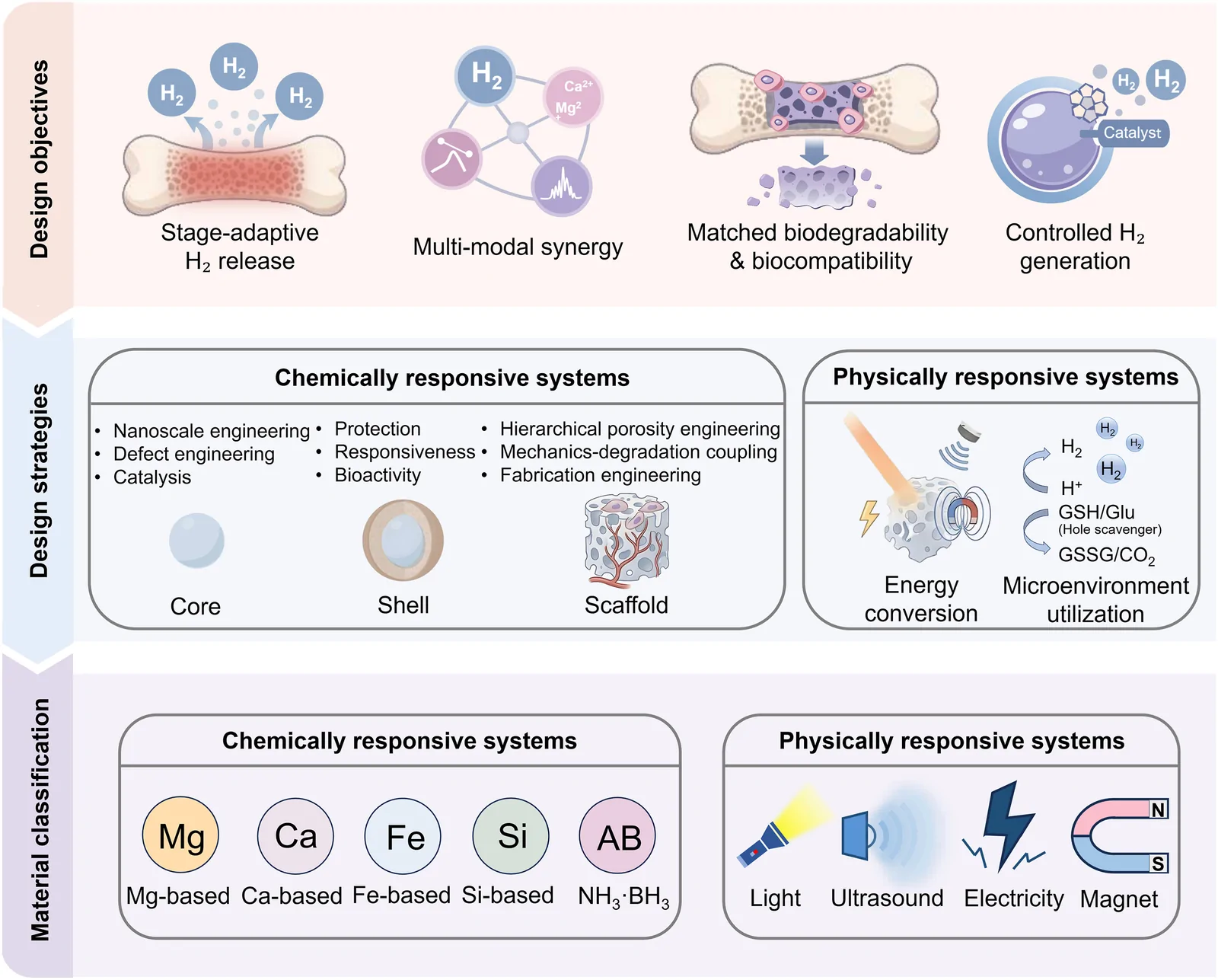
Overview of the design objectives, strategies, and classifications of H_2_-releasing biomaterials.

The pathological microenvironments associated with bone defects are highly complex, and the regulatory effects of H_2_ are multidimensional. Given this, H_2_-releasing biomaterials that aim to modulate the local microenvironment have become a pivotal strategy in biomedical research. The design objectives for an ideal H_2_-releasing biomaterial should include: (1) Designing H_2_ release kinetics that are appropriately matched to the evolving bone-healing microenvironment [19]. For instance, relatively sustained H_2_ release during the early inflammatory phase may help alleviate excessive oxidative stress and inflammation, followed by a gradual decline as tissue repair progresses. This represents a desirable design principle, although the optimal concentration windows and stage-specific release requirements remain to be established. Such a release profile may help reduce potential concerns associated with excessive or poorly controlled H_2_ accumulation, such as bubble formation and local alkalinization. (2) Constructing multimodal synergistic functions [100]. Integrating H_2_ modulation with ion release, material mechanical support, and bioactive signaling enables a four-dimensional synergy of “H_2_-ion-mechanics-biosignal”, thereby significantly improving the pathological microenvironment. (3) Optimizing material degradation behavior and interfacial compatibility [101]. This entails matching its degradation rate to the healing process of the surrounding tissue, ensuring the safe clearance of byproducts, and enhancing interface stability and biocompatibility through surface engineering techniques. (4) Developing mild and controllable H_2_ supply strategies [35]. Approaches such as nanoencapsulation, catalyst modification, and composite scaffolds can enable safe and controllable H_2_ release under physiological conditions, offering feasible pathways for clinical translation (Fig. 3).

### 4.2 Design strategies

The design of H_2_-releasing biomaterials is fundamentally governed by the interplay between material composition, structural characteristics, and stimulus-responsive behaviors, which together determine the underlying H_2_ release mechanisms and release kinetics. Based on the trigger source and reaction principles, these materials can be broadly categorized into chemically responsive systems and physically triggered systems. The former typically relies on the hydrolysis or corrosion of precursors (e.g., metals or metal hydrides) under physiological conditions, whereas the latter utilizes external energy inputs (e.g., light, ultrasound, or magnetic fields) to drive catalytic H_2_ generation. This section summarizes the core design strategies underlying these two categories.

#### 4.2.1 Chemically responsive systems

Chemically responsive H_2_-releasing systems predominantly utilize the intrinsic redox reactivity of metals or metalloids with low standard electrode potentials. Upon contact with aqueous media, these materials undergo spontaneous hydrolysis or corrosion-mediated reactions, reducing water molecules to generate H_2_. As reviewed by Ouyang et al., the H_2_ generation kinetics are governed primarily by the thermodynamic activity and intrinsic properties of the core materials [102]. Rational structural engineering is therefore essential to achieve controllable and sustained H_2_ delivery. To this end, hierarchical architectures comprising core, shell, and scaffold components have been developed as representative strategies. These strategies regulate reaction kinetics, improve microenvironmental responsiveness, and integrate multifunctional therapeutic properties. Although not universally required, such hierarchical designs currently represent the predominant approaches for achieving functional integration and controlled H_2_ release.

Core Design: As the central determinant of the H_2_ release mechanism, kinetics, and byproduct profile, its rational construction is crucial for developing safe and controllable H_2_-releasing materials. This is achieved through strategies such as particle size control, defect engineering, nanoconfinement, and catalyst incorporation, which collectively modulate reaction activation energy and rate. For example, magnesium hydride (MgH_2_) microspheres (with an average diameter of ∼20 μm) offered an optimized geometric surface that enhanced the availability of active sites for hydrolysis [103]; while layered magnesium silicide (Mg_2_Si) achieved a controllable H_2_ release window of 7-28 days by adjusting the thickness of its nanosheets [104]. Defect engineering, such as the introduction of oxygen vacancies, can enhance water molecule adsorption and modulate the interfacial configuration of active H_2_ sources. This enabled stable confinement of ammonia borane (AB) within hybrid-phased titanate nanocrystals (HyPT), achieving sustained H_2_ release for over 11 days [105]. Building upon this, nanoconfinement design, which confines the active H_2_ source within a carrier, effectively inhibits excessive hydrolysis and byproduct leakage. A typical example was the anchoring of AB within oxygen-deficient mixed-phase titanate nanocrystals, which not only prevented its migration and leakage but also avoided localized toxicity from synergistic effects with H_2_O_2_ [105]. In addition, introducing catalytic components can further reduce the reaction activation energy, enabling the material to efficiently release H_2_ under mild stimulation conditions and to regulate the H_2_ production rate to match tissue repair rhythms. For example, single-atom catalytic materials could provide a high density of active sites even at low loadings, significantly accelerating the hydrolytic dehydrogenation of AB, while their integration with the core structure may offer greater flexibility in modulating H_2_ release behavior [106]. Through these core design strategies, H_2_-releasing biomaterials with tunable release behaviors and improved biosafety can be rationally developed for bone repair applications.

Shell design: The shell layer serves as a reaction-regulation barrier in H_2_-releasing systems, determining the interface reaction rate between the core material and body fluid, H_2_ diffusion, byproduct isolation, and biocompatibility. Coating the core material with biodegradable polymers allows modulation of its hydrophilicity, degradation rate, and spatial structure [[107], [108], [109]], thereby enabling mild and controllable H_2_ release [38,103]. Based on characteristics such as pH, ROS, and the expression levels of specific enzymes in different pathological microenvironments, the shell material can be designed with various stimuli-responsive groups to achieve controlled release of H_2_. For example, the fabrication of Fe nanoparticles coated with carboxymethyl cellulose (CMC), denoted as Fe@CMC, enabled localized and targeted H_2_ release in response to pH gradients [110]. The shell layer can also serve as a carrier for bioactive molecules or therapeutic agents, enabling multimodal synergistic regulation. For instance, a polydopamine shell dual-modified with BMP-2/VEGF enhanced osteogenesis and angiogenesis [111], while a PLGA/calcium (Ca) phosphate bilayer shell incorporating paclitaxel promoted bone defect regeneration in the context of osteosarcoma resection and the associated bone defect [112]. At the same time, the shell layer also needs to effectively isolate or slowly release potential toxic by-products generated during the reaction through physical barriers, chemical complexation, or reaction buffering. This prevents their transient accumulation in local tissues, thereby reducing cytotoxicity and inflammatory risks [113].

Scaffold design: As the key platform connecting the core-shell structure with tissue regeneration, the scaffold provides mechanical support while modulating cell behavior, vascularization, and the overall quality of tissue reconstruction. Scaffold architecture also governs the coupling between degradation behavior and H_2_ release kinetics through solution transport processes and local microenvironment evolution. Such dynamic gas-solid interactions affect H_2_ diffusion and bubble retention, potentially leading to progressive deterioration of scaffold structural integrity during degradation [114]. At the same time, multiscale porous architectures facilitate gas diffusion, cell infiltration, and nutrient transport. For example, a 3D-printed PLGA/β-TCP scaffold (∼600 μm pore size) effectively promoted osteogenesis and angiogenesis through the synergistic interplay between the relatively rapid degradation of β-TCP and the sustained release behavior of PLGA [115]. However, in H_2_-releasing systems, continuous bubble formation and escape may alter internal porosity and disrupt pore integrity, leading to microstructural heterogeneity and mechanical instability, especially under load-bearing conditions. The gas-solid interaction accelerates structural evolution during degradation, thereby highlighting the need for control of pore architecture. Such progressive microstructural changes may consequently impair the long-term mechanical stability of the scaffold during degradation. Constructing highly interconnected and hierarchical porous structures with optimized porosity can facilitate gas diffusion and mass transport, thereby reducing gas accumulation, preserving pore interconnectivity, and mitigating mechanical instability under physiological conditions [116]. Furthermore, scaffolds should provide initial mechanical support matching native bone and undergo controlled degradation that synchronizes with new bone ingrowth to avoid stress shielding [117]. For instance, a SF/MgO composite scaffold enabled tunable compressive modulus and degradation by adjusting MgO content, thereby enhancing bone repair [118]. In this context, the coupling between mechanics, degradation, and gas evolution is critical. Pore evolution and bubble dynamics accelerate mechanical attenuation and reshape H_2_ release pathways, requiring coordinated regulation to maintain structural reliability and therapeutic efficacy. In addition, the introduction of advanced techniques such as electrospinning [119,120], biomimetic mineralization [121], and 4D bioprinting [122,123] can further endow the scaffold structure and function with self-adaptive regulatory capabilities, providing potential pathways for personalized bone repair and integration with intelligent H_2_-releasing systems.

It is worth noting that H_2_-releasing components can be integrated with scaffold systems through various conventional approaches, including surface functionalization, matrix encapsulation, or physical combination [38,124]. As this review primarily focuses on material design strategies, detailed fabrication methodologies are not further discussed here.

#### 4.2.2 Physically responsive systems

This category encompasses materials that generate H_2_ upon exposure to external physical stimuli such as light, ultrasound, or magnetic field. The design focus shifts from controlling hydrolysis to optimizing energy conversion efficiency and improving stimulus-responsive control over the reaction.

Design for enhanced energy conversion: For catalytic systems (e.g., photocatalysts, sonocatalysts), the key is to construct interfaces that efficiently convert physical energy into chemical energy for water splitting. A primary strategy involves building heterojunctions or Schottky junctions to promote the separation of photo- or sono-generated electron-hole pairs, thereby boosting H_2_ production efficiency. For example, in a Pt-Bi_2_S_3_ Schottky heterostructure, Fermi-level-driven electron transfer from Bi_2_S_3_ to Pt induces band bending and forms a Schottky barrier. This barrier suppresses electron backflow and improves charge separation, thereby enabling sustained sonocatalytic H_2_ evolution [125]. For photocatalytic semiconductors, constructing upconversion core-shell nanoparticles (e.g., UCNP@g-C_3_N_4_) that convert deep-penetrating near-infrared (NIR) light into visible light is crucial [126]. This allows the material to be excited within deep tissues. Furthermore, the introduction of cocatalysts or the creation of defects is an equally pivotal strategy. Noble metals such as Pt and Pd serve as efficient proton-reduction sites and electron traps [127,128], while non-precious or dual cocatalysts further optimize charge utilization [129,130]. Introducing defects (e.g., oxygen vacancies) tailors the electronic structure, enhances light absorption, facilitates carrier separation, and creates active sites for water adsorption and activation [131]. In contrast, non-catalytic magnetically responsive systems rely on eddy-current-induced heating under an alternating magnetic field to accelerate the intrinsic Mg-water reaction for controlled H_2_ generation [39].

Leveraging the pathological microenvironment: Smart design can turn pathological features into therapeutic advantages. In the Pt-Bi_2_S_3_ system, elevated glutathione (GSH) in tumors serves as a natural hole scavenger. Upon photocatalytic excitation, GSH, as a reducing agent, consumed photogenerated holes, suppressed electron-hole recombination, and thereby increased electron availability for H_2_ generation [125]. Meanwhile, depletion of intracellular GSH weakened the anti-oxidative defense of cancer cells, enhanced oxidative stress sensitivity, and improved therapeutic efficacy [132]. Similarly, photocatalytic systems for diabetic wounds, such as H_2_-doped titanium oxide (H-TiO_2_) nanorods or Bi/Bi_2_WO_6_/H-TiO_2_ Z-scheme heterojunctions, exploited the hyperglycemic microenvironment by using excess glucose as a reducing agent to scavenge holes and promote H_2_ production [133,134]. Simultaneous glucose consumption alleviated hyperglycemia-associated oxidative stress, chronic inflammation, and impaired cellular function, thereby facilitating wound healing [135]. This dual-function strategy simultaneously consumes pathogenic molecules and generates therapeutic H_2_, thereby modulating the pathological microenvironment and synergistically promoting bone regeneration.

In summary, chemically responsive systems employ a multi-layered “core-shell-scaffold” architecture to achieve controllable and sustained hydrolysis, while physically responsive systems focus on engineering efficient energy-conversion interfaces (via heterojunctions, cocatalysts, or defect engineering) or leveraging the pathological microenvironment for controlled H_2_ generation. Both strategies aim to deliver H_2_ in a controlled manner to modulate the pathological microenvironment, thereby creating favorable conditions for tissue regeneration and repair.

### 4.3 Classification

H_2_-releasing biomaterials can be classified into two main categories based on their triggering mechanisms: chemically responsive and physically responsive systems. The former relies on endogenous chemical signals within the pathological microenvironment to achieve passive H_2_ release, offering well-defined reaction pathways without the need for external stimulation. However, their H_2_ release kinetics are susceptible to fluctuations in the in vivo microenvironment. The latter, in contrast, actively triggers H_2_ release through exogenous physical stimuli, featuring remote controllability and deep tissue penetration, yet they depend on external stimulation and may increase the complexity of material design and operation. The following sections will provide a systematic overview of the representative material systems, H_2_ release mechanisms, and biomedical applications for both categories.

#### 4.3.1 Chemically responsive H_2_-releasing biomaterials

Chemically responsive H_2_-releasing biomaterials generate H_2_ in pathological microenvironments through chemical reactions such as hydrolysis or corrosion promotion. The following summarizes several representative types of these materials.

Mg-based materials: Due to their favorable biocompatibility, controllable hydrolysis-mediated H_2_ release, and bioactive degradation products, these materials have become a research focus in the field of chemically responsive-H_2_ generation. Mg and MgH_2_ react with water to generate H_2_ (Mg + 2H_2_O → Mg(OH)_2_ + H_2_, MgH_2_ + H_2_O → Mg^2+^ + H_2_ + OH^−^). Studies often employ strategies such as coating with polymers or forming hydrogel composites to modulate the reaction rate and achieve stable and sustained H_2_ release. Moreover, the Mg^2+^ and OH^−^ ions released during this process can synergistically regulate the local pH and immune microenvironment, establishing an integrated therapeutic system that combines H_2_ therapy with microenvironment modulation [[136], [137], [138]]. Furthermore, Mg-based compounds formed with non-metallic elements, such as magnesium boride (MgB_2_) and Mg_2_Si, can also release H_2_ efficiently through hydrolysis or acid-catalyzed reactions (MgB_2_ + 2H^+^ + 6H_2_O → Mg^2+^ + 2B(OH)_3_ + 4H_2_; Mg_2_Si + 5H_2_O → Mg_2_SiO_3_(OH)_2_ + 4H_2_). Currently, such materials are often fabricated as two-dimensional nanostructures or composite hydrogels, demonstrating well-controllable H_2_ release and significant therapeutic potential in models of tumor therapy and wound repair [139,140].

Ca-based materials: This category is primarily represented by calcium hydride (CaH_2_), which enables sustained H_2_ release via hydrolysis (CaH_2_ + 2H_2_O → Ca(OH)_2_ + 2H_2_), simultaneously generating Ca^2+^ and raising the local pH. Preclinical studies have demonstrated the anti-oxidative and tissue-protective effects of CaH_2_ as a solid H_2_ source in both in vitro and in vivo models [141]. Recent advances have further developed nano-CaH_2_ and its corresponding injectable or interventional delivery systems. These systems can simultaneously achieve H_2_ immunotherapy, tumor calcification induced by intracellular Ca^2+^ overload, and neutralization of the acidic tumor microenvironment (TME), offering a novel synergistic approach for cancer therapy [142].

Fe-based materials: Unlike Mg- or Ca-based materials that release H_2_ directly via hydrolysis, Fe-based materials rely on acidic conditions for H_2_ generation (Fe + 2H^+^ → Fe^2+^ + H_2_). This mechanism makes them particularly suitable for pathological microenvironments characterized by mild acidity. Research has focused on stabilizing zero-valent iron (Fe^0^) using polymer coatings or metal-organic frameworks (MOFs) to impart acid-responsive properties. This enables in situ triggered H_2_ release within the TME, which can be combined with chemotherapy or other therapeutic modalities, expanding the application scope of Fe-based H_2_-releasing materials in biomedicine [110,143].

Si-based materials: Theoretically, silicon (Si) can react with water to generate H_2_. However, its surface tends to form a dense SiO_2_ passivation layer, limiting the reaction's progression. To address this, researchers have developed Si-based nanostructures rich in Si-H bonds, such as hydrogenated silicon nanosheets and porous Si, establishing more efficient hydrolysis-mediated H_2_ release pathways (SiH_x_ + 3H_2_O → H_2_SiO_3_ + (2 + x/2)H_2_) [144]. These materials typically possess high specific surface areas and tunable reactivity. Their H_2_ release behavior can be modulated through nanoscale dimensions, surface modifications, and pH responsiveness. Recent studies have demonstrated their favorable targeting capability and biosafety in various inflammatory disease models [145,146].

AB-based materials: AB, a typical chemical hydride, represents a significant research focus in chemically responsive H_2_ generation systems due to its exceptionally high H_2_ capacity and acid-responsive H_2_ release behavior. Its H_2_ release reaction can be expressed as: NH_3_BH_3_ + 3H_2_O → NH_3_ + B(OH)_3_ + 3H_2_. Given the potentially rapid and difficult-to-regulate H_2_ release from AB under appropriate hydrolytic conditions and potential safety risks, current research often focuses on encapsulating AB within mesoporous structures or constructing core-shell nanocarriers. This allows for control of the release kinetics and triggering conditions, potentially enabling sustained H_2_ release in acidic pathological microenvironments such as the gastrointestinal tract or tumors. Furthermore, this approach exhibits significant synergistic therapeutic effects when combined with chemotherapy, photothermal therapy, or immunotherapy [147,148].

#### 4.3.2 Physically responsive H_2_-releasing biomaterials

Physically responsive H_2_-releasing biomaterials achieve remote triggering and stimulus-responsive regulation of H_2_ release by introducing external physical stimuli such as light or ultrasound. Based on the different mechanisms by which physical stimuli mediate H_2_ release, existing research can be mainly divided into two categories: light-responsive and ultrasound-responsive.

Light-responsive materials: In light-responsive H_2_-releasing biomaterials, researchers employ external optical signals to achieve remote and controllable control over H_2_ release. Based on their operational mechanisms, such materials are primarily categorized into photothermally responsive and photocatalytic types. Photothermally responsive materials absorb light energy at specific wavelengths and efficiently convert it into localized heat. This heat subsequently induces the cleavage of chemical bonds or facilitates the physical desorption of H_2_ from the material, enabling light-triggered H_2_ release. Palladium hydride (PdH) nanomaterials are a representative system within this category. They achieve high-density H_2_ storage by embedding hydrogen atoms into the palladium lattice to form stable hydrides. Under NIR light irradiation, the photothermal effect triggers the dissociation of Pd-H bonds, thereby regulating H_2_ release kinetics. Studies have demonstrated that PdH systems enable tunable H_2_ release kinetics through adjustment of irradiation parameters, which has shown synergistic therapeutic potential when combined with photothermal therapy or metal-ion-mediated antibacterial effects in the treatment of infections and tumors [[149], [150], [151], [152]].

Photocatalytic materials, in contrast, rely on semiconductor materials to generate photogenerated electron-hole pairs under illumination. These charge carriers drive the decomposition of water or other endogenous substrates to produce H_2_. Typical materials include graphitic carbon nitride (g-C_3_N_4_), photoactive MOFs, and titanium dioxide (TiO_2_). For instance, g-C_3_N_4_, a non-metallic semiconductor polymer, possesses photocatalytic H_2_ production capability, but its photo-response is mainly limited to the UV-visible range, which restricts its in vivo application. To address this, researchers have developed strategies to couple it with upconversion nanoparticles (UCNPs) to extend its light absorption range into the NIR region and enhance solar spectrum utilization [153]. Ge et al. demonstrated that by introducing UCNPs and co-catalysts (e.g., CoP), highly penetrative NIR light could be effectively converted into excitation signals for g-C_3_N_4_ [126]. This enables non-invasive, sustainable, and light-controlled H_2_ production using endogenous water molecules as the H_2_ source under NIR irradiation (2H_2_O → 2H_2_ + O_2_). Beyond serving as material carriers, some photoactive MOFs with well-defined valence/conduction band structures, such as Ti-based MOFs, can directly participate in the photocatalytic H_2_ evolution reaction [154,155]. Their photocatalytic performance is often further enhanced by introducing co-catalytic metals, constructing heterojunctions, or coupling with UCNPs. In addition, TiO_2_-based materials constitute another important class of photocatalytic H_2_-releasing systems. Recent advances, including H_2_ doping [156], metal cocatalyst loading [157] and heterostructure engineering, have significantly enhanced their light response and H_2_ evolution efficiency. Studies have begun to introduce TiO_2_-based photocatalytic H_2_ systems into disease treatment, utilizing endogenous water and glucose as reaction substrates to achieve sustained H_2_ generation under light irradiation (H_2_O + glucose → H_2_ + gluconic acid), while simultaneously modulating the hyperglycemic pathological microenvironment [133,134].

Ultrasound-responsive materials: Ultrasound-responsive H_2_-releasing materials leverage the non-invasiveness, deep penetration, and controllability of external ultrasound, providing a strategy for controlled H_2_ delivery. Based on their release mechanisms, these systems can be broadly classified into two categories: carrier-type materials based on physical encapsulation with ultrasound-triggered release and catalytic-type materials that generate H_2_ through ultrasound-mediated catalytic reactions. A representative carrier-type system is the phospholipid microbubble-based H_2_ delivery platform (H_2_-MBs) developed by He et al. [158]. In this system, H_2_ is co-encapsulated with a stable gas like C_3_F_8_ within a phospholipid bilayer to form H_2_-loaded microbubbles. Under ultrasound irradiation, the microbubbles oscillate and rupture, enabling localized H_2_ release. Simultaneously, the process allows real-time visualization via ultrasound imaging. This strategy has demonstrated therapeutic potential in models of myocardial ischemia-reperfusion injury. Catalytic-type materials are exemplified by the Pt-Bi_2_S_3_ Schottky heterostructure nanomaterials designed by Yuan's team [125]. This material does not contain pre-stored H_2_. Instead, upon ultrasound excitation, the semiconductor (Bi_2_S_3_) generates electron-hole pairs, which are separated via the heterojunction. This process utilizes water for in-situ and sustained catalytic H_2_ generation, while simultaneously consuming reducing substances in the tumor microenvironment (such as GSH), demonstrating advantages in synergistic sonodynamic-H_2_ therapy against tumors.

In addition to the two major systems mentioned above, the emergence of materials responsive to electric fields, magnetic fields, and piezoelectric effects is continuously expanding the scope of physically responsive H_2_-releasing materials. For instance, electric field-responsive manganese-doped Ni_2_S_3_ nanoelectrodes leverage their efficient electrocatalytic H_2_ evolution activity to achieve controlled H_2_ release at tumor sites under an applied electric field [159]. Magnetothermal-responsive Mg implants, under the influence of an alternating magnetic field (AMF), exhibit significantly accelerated corrosion kinetics via eddy current thermal effects and enable controlled H_2_ release [39]. Meanwhile, piezoelectric-responsive nanomaterials such as GDYO@Pt [40] and tetragonal barium titanate nanoparticles [160] catalyze water splitting under ultrasonic excitation via the piezoelectric effect, also achieving controlled H_2_ release.

## 5 Current status of H_2_-releasing biomaterials in bone regeneration

As the multifaceted biological effects of H_2_ are progressively elucidated, its application in bone defect repair has receiving increasing attention. During the early exploratory phase, H_2_ therapy relied on traditional exogenous administration methods such as H_2_ inhalation [29], oral intake [[31], [32], [33]], or injection of H_2_-rich water [161]. However, these approaches face significant limitations, including imprecise dosage control, lack of targeting capability, and poor spatiotemporal regulation of release. Consequently, the development of biomaterials capable of achieving sustained and controllable H_2_ release within the bone defect microenvironment has become an important direction in bone tissue engineering.

To provide a balanced overview of the rapidly evolving field of H_2_-releasing biomaterials for bone regeneration, relevant studies were identified through searches of PubMed and Google Scholar using combinations of keywords including hydrogen/H_2_, molecular hydrogen, H_2_-releasing biomaterials, hydrogen delivery systems, bone regeneration, bone repair, bone tissue engineering, osteogenesis, and related terms. Only peer-reviewed articles published in English were included in the literature search. The literature search was initially run on December 1, 2025, and rerun on July 31, 2026, to capture newly published studies during the revision process. No restrictions were applied regarding publication dates. Studies included in Table 1 were selected based on their relevance to H_2_-releasing biomaterial strategies for the broader skeletal regenerative field. All these studies were published within the past five years, reflecting the recent emergence of this research area. The selected studies cover critical-sized bone defects [38,40,41,162], infection-associated bone defects [39,163,164], osteoporosis-related bone defects [36,37,165,166], diabetes-associated bone defects [42,103,105] and osteoarthritis (OA) [34,43,104,160,[167], [168], [169]]. Studies on OA were included because OA involves remodeling of the osteochondral unit, and shares several H_2_-responsive biological processes with bone regeneration, including oxidative stress, inflammation, and bone remodeling. Therefore, these studies provide complementary evidence for evaluating the therapeutic potential of H_2_-releasing biomaterials in skeletal tissue regeneration. The following sections systematically summarize H_2_-releasing biomaterials for bone regeneration reported in recent years, with a particular focus on material design, H_2_-generation mechanisms, and their biological functions (Table 1).

**Table 1:** Summary of applications of H_2_-releasing biomaterials in bone regeneration.

Core	Shell	Scaffold	H_2_ release mechanism	H_2_ release duration	Disease model	Overall therapeutic effect (vs. control)^a^	H_2_ contribution (vs. non-H_2_)^b^	Refs
Chemically responsive H_2_-releasing biomaterials								
Mg spheres (50 μm)	PEG	PLGA	Mg + 2H_2_O → Mg(OH)_2_ + H_2_↑	7 d (in vitro)	Osteoporotic femoral defects in OVX rats	BV/TV: ∼2-fold (8 w)^c^	BV/TV: ∼1.54-fold (8 w)^d^	[36]
Mg spheres	QP-P		Mg + 2H_2_O → Mg(OH)_2_ + H_2_↑	Over 4 d (in vitro)	*S. aureus*-induced alveolar bone defect in rats	BV/TV: ∼5.02-fold (6 w)^c^	NA^f^	[163]
Mg spheres (20 μm)	GelMA		Mg + 2H_2_O → Mg(OH)_2_ + H_2_↑	28 d (in vitro)	Osteoporotic cranial defects in OVX mice	BV/TV: ∼1.29-fold (8 w)^d^	NA^f^	[37]
Mg spheres	GelMA		Mg + 2H_2_O → Mg(OH)_2_ + H_2_↑	28 d (in vitro)	Osteoporotic cranial defects in OVX mice	BV/TV: ∼1.42-fold (8 w)^d^	NA^f^	[165]
MMW	MPN	Collagen	Mg + 2H_2_O → Mg(OH)_2_ + H_2_↑	28 d (in vitro); 14 d (in vivo)	Cranial defects in rats	BV/TV: ∼2.13-fold (8 w)^d^	NA^f^	[162]
MgH_2_ spheres (19.1 μm)	PLGA	F-GM	MgH_2_ + 2H_2_O → Mg(OH)_2_ + 2H_2_↑	Over 15 d (in vitro)	Cranial defects in diabetic mice	BV/TV: ∼2.49-fold (12 w)^c^	NA^f^	[103]
MSNs	GelMA/HAMA hydrogel		Mg_2_Si + 5H_2_O → 2Mg^2+^ + SiO_3_^2−^ + 4H_2_↑ + 2OH^−^	28 d	Osteoarthritic trochlear cartilage defects in rats	OARSI Score: ∼1.23 (control: ∼4)^d^	OARSI Score: ∼1.23 (non-H_2_: ∼3.02)^d^	[104]
CBN	GelDA		3CaB_6_ + 54H_2_O →Ca_3_(BO_3_)_2_ + 16H_3_BO_3_ + 30H_2_↑	28 d (in vitro)	MIA-induced osteoarthritic knee joint in rats	BV/TV: ∼1.31-fold (4 w)	NA^f^	[167]
						Mankin Score: ∼4.65 (vs. ∼10 in control)^d^		
CaH_2_			CaH_2_ + 2H_2_O → Ca(OH)_2_ + H_2_↑	NR^g^	*S. aureus*-induced tibial osteomyelitis in mice	BV/TV: ∼2.32-fold (4 w)^d^	BV/TV: ∼1.37-fold (4 w)^d^	[164]
CSN	PHA	MBG	CaSi_2_ + 2OH^−^ + 4H_2_O → Ca^2+^ + 2SiO_3_^2−^ + 5H_2_↑	7 d (in vitro); 9 d (in vivo)	Critical-size femoral plug defects in aged (24-month-old) mice	BV/TV: ∼2.17-fold (4 w)^d^	BV/TV: ∼1.49-fold (4 w)^d^	[38]
nSi	RA-MC fibers		Si + 4H_2_O → Si(OH)_4_ + 2H_2_↑	3 d (pH = 7.4); 7 d (pH = 6.5)	Full-thickness calvarial defects (4 mm) in mice	BV/TV: ∼68.12% vs. near-zero control (8 w)^d^	NA^f^	[41]
H-Si nanosheets	GelMA short fibers		2SiH + 4OH^−^ + 2H_2_O → 2SiO_3_^2−^ + 5H_2_↑	4 d (in vitro)	MIA-induced osteoarthritic knee in rats	Inconsistent OARSI Score	NA^f^	[34]
MSC-PPA			Acid-triggered AB hydrolysis	NR^g^	Modified Hulth's method-induced osteoarthritic knee in rats	BV/TV: ∼1.35-fold (8 w)^c^	BV/TV: ∼1.15-fold (8 w)	[43]
						OARSI Score: ∼1.33 (control: ∼4.68)^d^	OARSI Score: ∼1.33 (non-H_2_: ∼3.16)^d^	
mPDA@Mel-AB	OCS/CMC-BG		Acid-triggered AB hydrolysis	NR^g^	MIA-induced osteoarthritic knee in rats	BV/TV: ∼1.81-fold (4 w)	NA^f^	[168]
						OARSI Score: ∼3.38 (control: ∼15.1)^d^		
Se-HMPB@AB nanocomposites	COS		Acid-triggered AB hydrolysis	NR^g^	ACLT-induced osteoarthritic knee in mice	BV/TV: ∼1.52-fold (8 w)^e^	NA^f^	[169]
						OARSI Score: ∼4.08 (control: ∼10.19)^d^		
AB	HyPT		AB hydrolysis	11 d (in vitro)	Critical-size bone defects in diabetic rabbits	BV/TV: ∼5-fold (4 w)^d^	BV/TV: ∼1.77-fold (4 w)^d^	[105]
Physically responsive H_2_-releasing biomaterials								
g-C_3_N_4_ nanosheets	PDA	Fg	Photocatalytic water splitting	Externally triggered^h^	Calvarial defects in diabetic aged rats	BV/TV: ∼4.1-fold (8 w)^e^	NA^f^	[42]
GDYO@Pt	TA-modified poloxamer thermosensitive gel		US-triggered piezoelectric H_2_ release	Externally triggered^h^	Full-thickness cranial defects (3 mm) in mice	BV/TV: ∼9.74-fold (12 w)^d^	BV/TV: ∼2.82-fold (12 w)^d^	[40]
MSN@BTO	MnO_2_		US-triggered piezoelectric H_2_ release	Externally triggered^h^	MIA-induced osteoarthritic knee in rats	BV/TV: ∼1.43-fold (3 w)	BV/TV: ∼1.26-fold (3 w)	[160]
						OARSI Score: ∼2.03 (control: ∼6.38)^d^	OARSI Score: ∼2.03 (non-H_2_: ∼4.54)^d^	
Mg rods			Eddy-thermal-activated Mg hydrolysis	Externally triggered^h^	*S. aureus*-induced tibial osteomyelitis in mice	BV/TV: ∼2.63-fold (4 w)^d^	NA^f^	[39]
Pd nanocubes	ZIF-8	PAA	Physical desorption/diffusion	NR^g^	Osteoporotic bone in OVX mice	BV/TV: ∼2.55-fold (12 w)^e^	BV/TV: ∼1.38-fold (12 w)^d^	[166]

**Abbreviations:** QP-P, the double-network hydrogel composed of phenylboronic acid-modified quaternary chitosan and glycidyl methacrylated poly (vinyl alcohol); MMW, micro-Mg wire; MPN, metal-phenolic network; MSNs, Mg_2_Si nanoplates; CBN, Ca boride nanosheets; GelDA, dopamine-grafted gelatin hydrogel; MIA, Monoiodoacetate; CSN, CaSi_2_ nanoparticles; nSi, nanosilicon; RA-MC fibers, radially aligned mineralized collagen fibers; MSC-PPA, MSCs conjugated with Polydopamine-PEG-Ammonia Borane Nanoparticles; mPDA@Mel-AB, mesoporous dopamine nanoparticles loaded with melatonin and ammonia borane; OCS/CMC-BG, the injectable hydrogel is composed of oxidized chondroitin sulfate and carboxymethyl chitosan, cross-linked via dynamic Schiff base reaction, and integrated with bioactive glass (BG); Se-HMPB@AB, selenium-coated hollow mesoporous Prussian blue loaded with ammonia borane; COS, Chondroitin Sulfate; HyPT, Hybrid Phased Titanate Crystal Surface; Fg, fibrin hydrogel; GDYO@Pt, platinum-decorated graphdiyne oxide; TA, tannic acid; MSN@BTO, barium titanate nanoparticle-loaded mesoporous silica nanoparticles; ZIF-8, zeolitic imidazolate framework-8; PAA, poly(acrylic acid).

Notes:

a Control denotes the untreated group.

b Comparison between H_2_-releasing systems and corresponding non-H_2_-releasing counterparts with otherwise identical material compositions.

c Fold change reported directly in the original publication.

d Fold change calculated from digitized graphical data extracted using WebPlotDigitizer 5.2 when numerical values were unavailable, values should be interpreted as approximate.

e Fold change calculated from numerical values reported in the original publications.

f Not available because the original studies lacked appropriate composition-matched non-H_2_ controls.

g Not reported in the original studies.

h For physically responsive systems, H_2_ generation is governed by external stimulation and therefore cannot be represented by a fixed release duration.

### 5.1 Application of chemically responsive H_2_-releasing biomaterials in bone regeneration

#### 5.1.1 Mg-based H_2_-releasing biomaterials

Mg-based materials have gained extensive attention in bone tissue engineering due to their excellent biocompatibility, controllable degradation, and inherent osteogenic activity [170]. Centered on their hydrolysis-mediated H_2_ release properties, related research has evolved from early single-component Mg particle systems toward multilevel, multifunctional composite architectures. H_2_ release sources now include metallic Mg, MgH_2_, and Mg_2_Si.

**Fig. 4:**
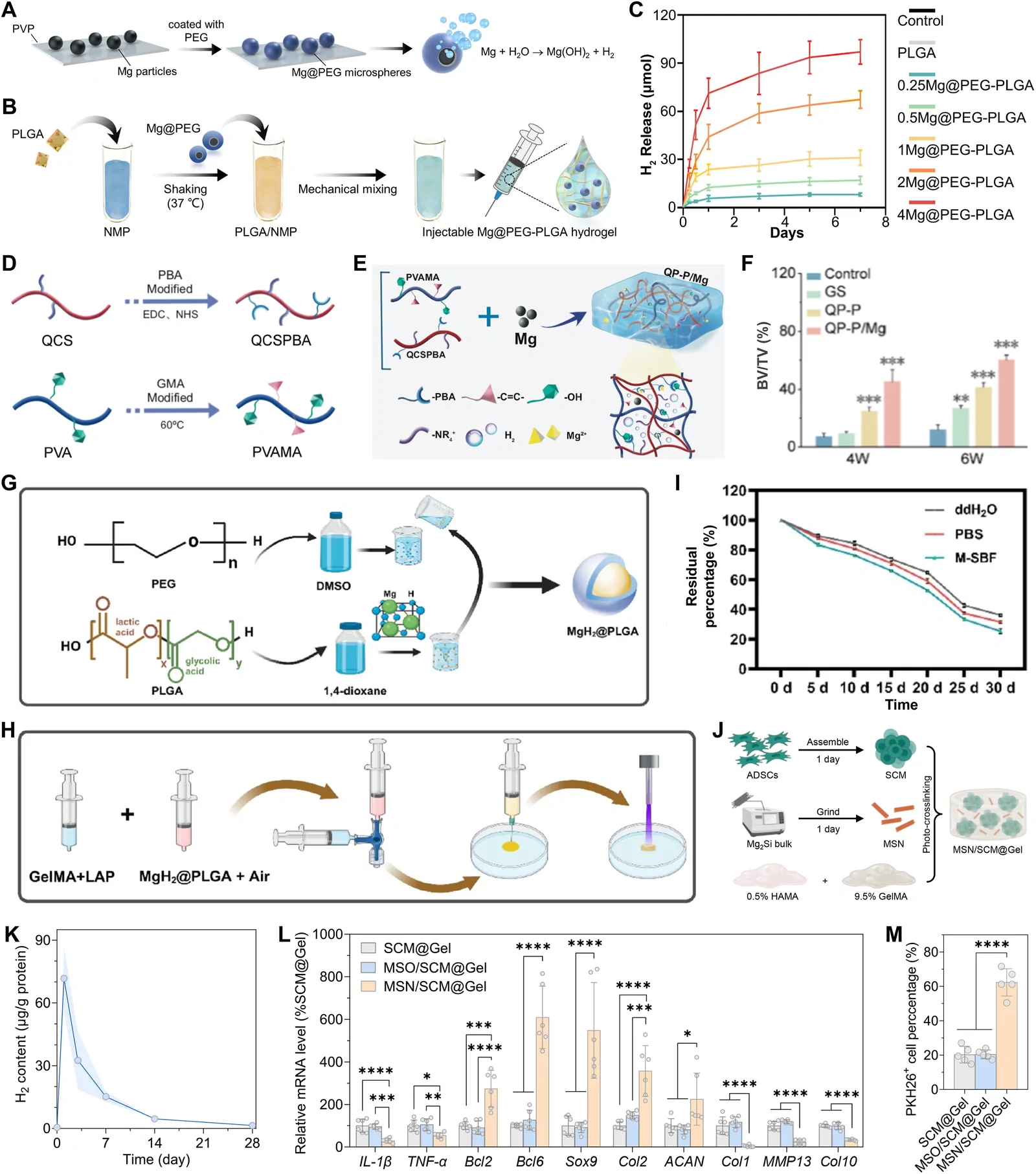
Representative studies of Mg-based H_2_-releasing biomaterials for bone regeneration. (A) Schematic diagram of the preparation of Mg@PEG microspheres. (B) Schematic diagram of the preparation of the Mg@PEG-PLGA hydrogel. (C) Time-dependent H_2_ generation measured by a methylene blue (MB) probe from Mg@PEG-PLGA hydrogel. Reproduced with permission [36]. Copyright 2024, The authors. (D) Synthesis of phenylboronic acid-modified quaternized chitosan (QCSPBA) and methacrylated polyvinyl alcohol (PVAMA); (E) Schematic diagram of the preparation of QP-P/Mg. (F) Semiquantitative analysis of bone volume/tissue volume (BV/TV). Reproduced with permission [163]. Copyright 2025, The authors. (G) Fabrication schematic of MgH_2_@PLGA microspheres. (H) Schematic of the foamed GelMA (F-GM) preparation process. (I) Comparative analysis of the degradation rates of F-GM, MgH_2_/F-GM, and MgH_2_@PLGA/F-GM over a 30-day period. Reproduced with permission [103]. Copyright 2024, American Chemical Society (ACS). (J) The method of MSN/SCM@Gel preparation; (K) The H_2_ release behavior of MSN@Gel in vivo. (L) Gene expression analysis of neo-tissue at the defect site. (M) Quantitative analysis of PKH26 cells in the newly formed tissue at the defect site. Reproduced with permission [104]. Copyright 2025, Elsevier.

Currently, most studies utilize metallic Mg as the H_2_-releasing core. For example, Zhou et al. prepared 2 Mg@PEG-PLGA hydrogels by coating Mg particles with asymmetric polyethylene glycol (PEG) (Fig. 4A and B). This hydrogel enables staged H_2_ release: an initial burst release for early ROS scavenging, followed by a sustained, slow release lasting up to 7 days to support the remodeling of the bone repair microenvironment (Fig. 4C). In an OVX rat femoral defect model, it enhanced the bone volume fraction (BV/TV) by more than twofold compared with the control group at 8 weeks post-implantation, resulting in nearly complete defect healing [36]. Kong et al. dispersed Mg microspheres in a mixed solution of methacrylated polyvinyl alcohol (PVAMA) and phenylboronic acid-modified quaternized chitosan (QCSPBA) (Fig. 4D). After rapid crosslinking via dynamic boronate ester bonds and subsequent UV curing, they fabricated a dual-network hydrogel (QP-P/Mg) (Fig. 4E). The dynamic boronate ester bonds modulated the H_2_ release from Mg hydrolysis. In a rat model of infectious mandibular bone defect, this system played multiple roles in scavenging ROS and activating the Nrf2/HO-1 pathway to repair mitochondria, resulting in a BV/TV of 60.18% at 6 weeks post-defect, approximately five times that of the control group (Fig. 4F), along with significantly enhanced bone mineral density (BMD), trabecular number (Tb.N), and trabecular thickness (Tb.Th) [163]. Other studies have introduced synergistic strategies that incorporate release drugs. For example, Cao et al. developed a multifunctional Gel-Mg-ALN microsphere scaffold (GMA MSs), which provided early anti-inflammatory effects through H_2_ release, followed by sustained release of alendronate sodium (ALN) and Mg^2+^. This system significantly promoted bone regeneration compared with untreated controls in osteoporotic skull defects of OVX mice [37]. Similarly, Lu et al. designed a pH-responsive GelMA/Mg/DMF microsphere scaffold by co-encapsulating Mg microspheres with the pyroptosis inhibitor dimethyl fumarate (DMF). In acidic, inflammation-related microenvironments, rapid H_2_ release delivered early anti-oxidative and anti-inflammatory effects, while staged Mg^2+^ and DMF release inhibited pyroptosis and promoted osteogenesis. This resulted in nearly complete repair of 2 mm calvarial defects in OVX mice 8 weeks post-implantation [165]. Recent research further expands the dimension of bone immune-metabolic regulation of H_2_-releasing Mg materials. Mu et al. embedded metal-phenolic network (MPN)-coated micro-Mg wires into a bi-layered collagen membrane (Col-MMW), enabling controlled release of H_2_ and Mg^2+^ through controlled degradation. Its uniqueness lies in its ability to regulate osteoimmunometabolism by shifting macrophage metabolism from glycolysis towards oxidative phosphorylation, activating the Nrf2/HO-1 anti-oxidative pathway, and inhibiting the NF-κB inflammatory pathway, thereby significantly promoting bone regeneration in a rat calvarial defect model. Notably, at the cellular level, the Col-MMW group exhibited enhanced RUNX2-positive signals compared to the Col-alone group. This suggests that Col-MMW may more effectively promote osteogenic differentiation, likely through its immunomodulatory effects [162].

In Mg hydride-based systems, Pei and co-workers encapsulated MgH_2_ microspheres within PLGA to fabricate an injectable porous scaffold (MgH_2_@PLGA/F-GM) via gas foaming combined with 405 nm photo-crosslinking (Fig. 4G and H). The scaffold exhibited controllable degradation in simulated body fluid, reaching a degradation rate of 74.72% within 30 days (Fig. 4I), which closely matched the early inflammatory phase of bone defects and ensured sustained release of H_2_, Mg^2+^, and other therapeutic factors during the critical repair window. In a diabetic mouse model of cranial bone defects, the proportion of the defect area filled with new bone in the MgH_2_@PLGA/F-GM group was significantly higher than that in the control group after 12 weeks of treatment [103]. Furthermore, Chen et al. developed an injectable hydrogel based on Mg_2_Si (Fig. 4J). By co-encapsulating Mg_2_Si nanosheets with adipose-derived stem cell microspheres, the system enabled localized in vivo H_2_ release for up to 28 days (Fig. 4K). In a rat model of OA-related cartilage defect, this approach enhanced the survival of transplanted stem cells and maintained their chondrogenic differentiation potential (Fig. 4L and M), ultimately promoting cartilage tissue regeneration and joint function recovery [104].

Overall, Mg-based H_2_-releasing materials are evolving from single-function systems toward multimodal integrated regulation. This progression provides advanced material strategies for addressing complex bone defects.

#### 5.1.2 Ca-based H_2_-releasing biomaterials

**Fig. 5:**
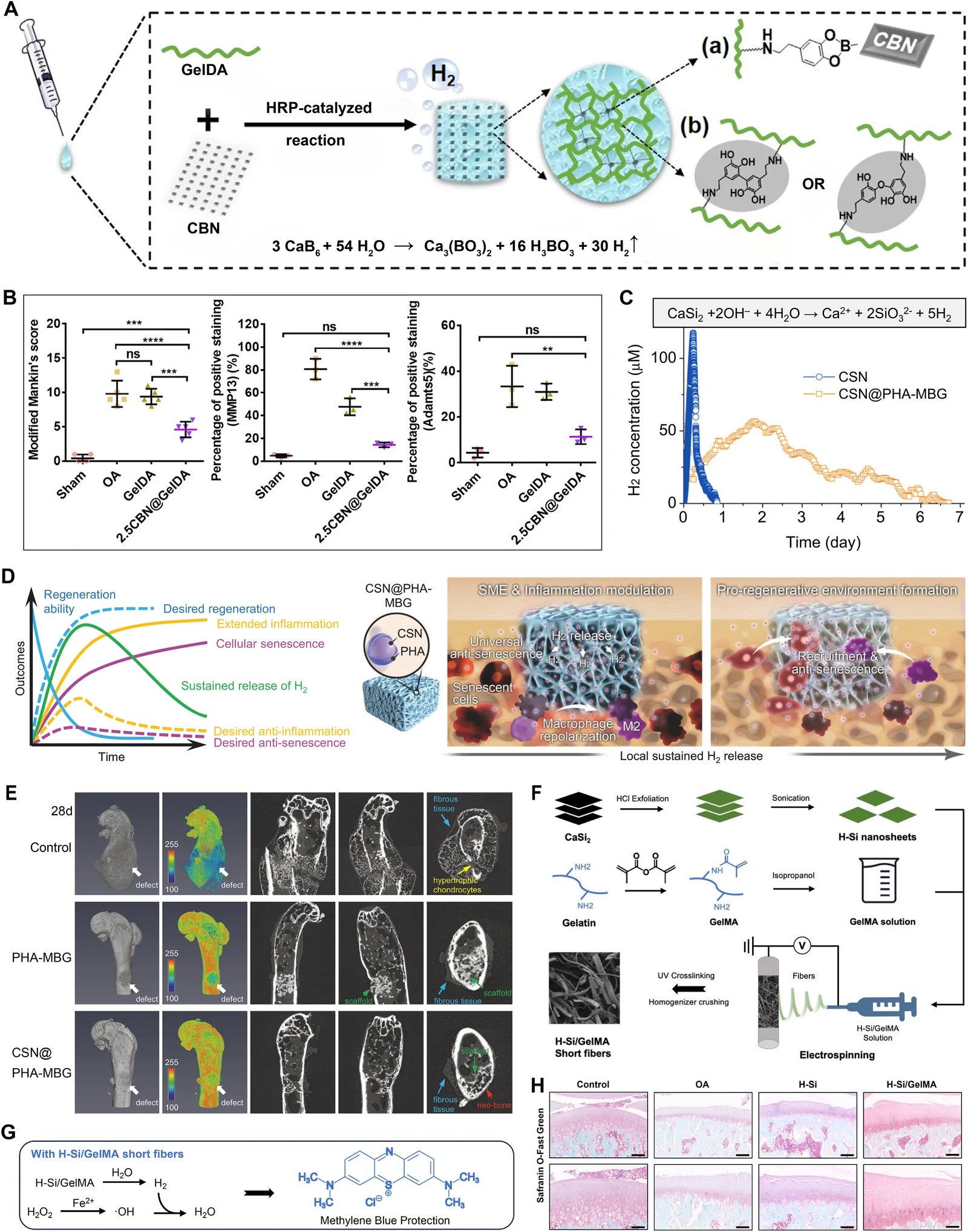
Representative studies of Ca-based and Si-based H_2_-releasing biomaterials for bone regeneration. (A) Schematic illustration showing the fabrication of the CBN@GelDA hydrogel. (B) Mankin's scoring of histopathological analysis (n = 5), the percentages of MMP13 and Adamts5 positive chondrocytes (n = 3). Reproduced with permission [167]. Copyright 2022, Elsevier and Acta Materialia. (C) The insert shows the reaction of CSN in the aqueous solution with a high H_2_ yield. (D) Schematic illustration of CSN@PHA-MBG scaffold-mediated sustained local H_2_ release for aged bone regeneration. (E) Representative Synchrotron Radiation Micro-Computed Tomography (SRμCT) images of neo-tissues at day 28 after Control, PHA-MBG or CSN@PHA-MBG treatment. Reproduced with permission [38]. Copyright 2023, The authors, published by Springer Nature. (F) Schematic diagram of H-Si/GelMA short fiber preparation. (G) Schematic diagram of the reaction system with H-Si/GelMA short fibers exerting anti-oxidative effects. (H) Safranin O-Fast Green staining; Scale bar: upper row 200 μm, lower row 400 μm. Reproduced with permission [34]. Copyright 2024, Acta Materialia.

Ca-based materials, such as hydroxyapatite and Ca phosphate ceramics, represent one of the primary material systems for bone defect repair. This is attributed to their highly similar composition to natural inorganic bone, excellent biocompatibility, and favorable osteoconductivity [171]. In recent years, research on converting them into H_2_-releasing functional materials has gradually emerged. Zhang et al. developed an injectable hydrogel (CBN@GelDA) based on Ca boride nanosheets (CBN) and dopamine-functionalized gelatin (GelDA) (Fig. 5A), which enabled sustained H_2_ release for over one week under physiological conditions. The hydrogel demonstrated significant anti-inflammatory and chondroprotective effects in a rat OA model (Fig. 5B) [167]. Another study constructed a “hydrogen/alkali/calcium” triple synergistic therapeutic system based on the multiple biological effects of CaH_2_ hydrolysis. In this system, in situ H_2_ release enhanced bacterial membrane permeability, massive Ca^2+^ influx induced bacterial Ca overload, and the simultaneous generation of OH^−^ exerted rapid bactericidal activity. In a *Staphylococcus aureus*-infected mouse tibial osteomyelitis model, this strategy demonstrated potent efficacy. After 28 days of treatment, bone marrow cultures from the CaH_2_-treated group showed nearly undetectable bacteria. Moreover, micro-CT analysis revealed that the BV/TV approached that of the uninfected sham group [164]. In addition, Chen et al. designed a composite scaffold consisting of polyhydroxyalkanoate (PHA)-coated Ca disilicide nanoparticles (CSN) supported on mesoporous bioactive glass (MBG), referred to as CSN@PHA-MBG. The scaffold achieved sustained H_2_ release for up to one week under physiological conditions (Fig. 5C) and remodeled the aging microenvironment in vitro and in aged mouse bone defect models (Fig. 5D), thereby effectively promoting bone regeneration (Fig. 5E) [38]. Collectively, Ca-based H_2_-releasing materials are evolving towards intelligent therapeutic platforms that integrate multiple functions, including antibacterial, anti-inflammatory, anti-oxidative, and osteogenic properties.

#### 5.1.3 Si-based H_2_-releasing biomaterials

The traditional function of Si-based materials in bone tissue repair mainly relies on the sustained release of osteogenic ions such as SiO_4_^4−^ and PO_4_^3−^ to promote bone matrix mineralization and facilitate tight integration between bone and the material [172]. In recent years, researchers have further integrated its H_2_-releasing properties to impart new bioactivity to Si-based materials. Mac et al. designed a radially aligned mineralized collagen scaffold loaded with nanosilicon (RA-MC/nSi scaffold), in which a silica layer gradually formed on the nSi surface during reaction with water in inflammatory microenvironments, thereby reducing the reaction rate and enabling sustained H_2_ release for approximately seven days. Beyond the biological mechanisms associated with H_2_, the silicic acid released from the scaffold degradation can also promote the proliferation of pre-osteoblasts and their subsequent differentiation into mature osteoblasts [41]. This combined effect was directly reflected in the significantly enhanced bone regeneration observed in vivo. At 8 weeks, the RA-MC/nSi scaffold achieved a BV/TV of approximately 70%, representing a 14-fold increase over the control group. These results quantitatively demonstrate the superior potential of this scaffold for treating large bone defects [41]. In addition, Pang and colleagues developed an injectable short-fiber system composed of electrospun hydride silicon (H-Si) nanosheets and GelMA (Fig. 5F), in which the controlled degradation of the GelMA matrix regulated the hydrolysis kinetics of H-Si, resulting in sustained anti-oxidative effects (Fig. 5G) and enhanced cartilage repair in a rat OA model (Fig. 5H) [34]. Through diverse material structural designs, these studies have expanded the application dimensions of Si-based H_2_-releasing materials in bone defect repair.

#### 5.1.4 AB-based H_2_-releasing biomaterials

**Fig. 6:**
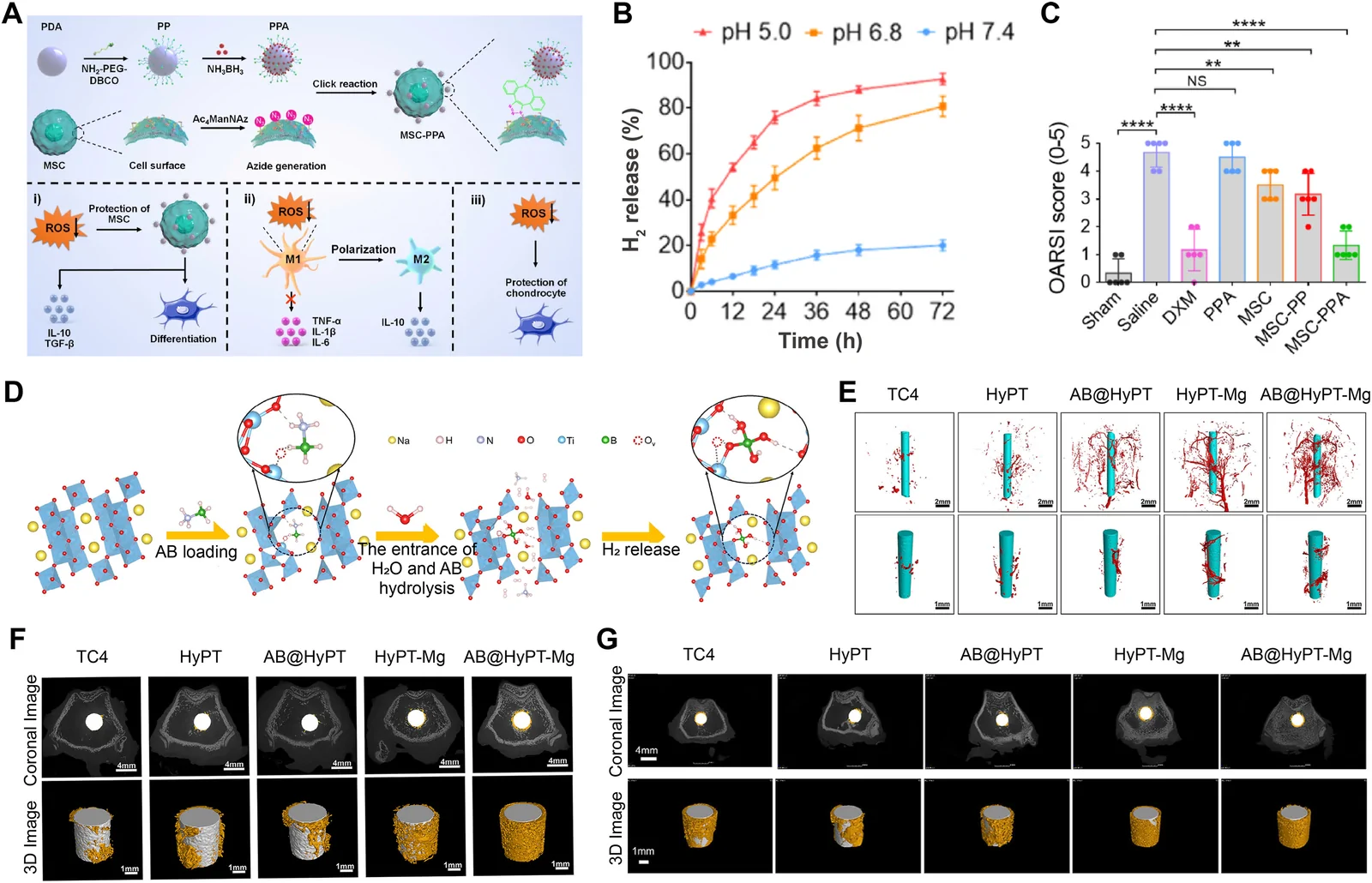
Representative studies of AB-derived H_2_-releasing biomaterials for bone regeneration. (A) Schematic diagram of the preparation and mechanism of MSC-PPA conjugates. (B) H_2_ release curve of PPA NPs at pH 5.0, 6.8, and 7.4. (C) Osteoarthritis Research Society International (OARSI) score of cartilage degeneration based on Toluidine Blue (TB)-stained sections. Reproduced with permission [43]. Copyright 2025, Elsevier. (D) Simulation diagram of the H_2_ release process of AB@HyPT: water molecules enter the interlayers of HyPT, causing AB to undergo hydrolysis and releasing H_2_, while borate remains within the interlayers. (E) 3D reconstructed micro-CT images of vascular distribution around the femoral distal end and implant two weeks after implantation in the normoglycemic rat bone defect model. (F) The ability of AB@HyPT-Mg to promote in vivo bone regeneration in a rabbit diabetic bone defect model. (G) The ability of AB@HyPT-Mg to promote in vivo bone regeneration in a rabbit normoglycemic bone defect model. Reproduced with permission [105]. Copyright 2025, Wiley-VCH GmbH.

AB has garnered significant interest as a H_2_-donating carrier in bone tissue engineering, owing to its high H_2_ storage capacity [173] and ability to release H_2_ under mild conditions. Through the integration of nanocarriers and smart-responsive strategies, AB-based systems enable targeted delivery and controlled H_2_ release within the pathological microenvironment, thereby simultaneously exerting multiple functions including ROS scavenging, immune regulation, and tissue regeneration. For example, Xu et al. constructed a cell-nanoparticle complex (MSC-PPA) by anchoring AB-loaded PEGylated polydopamine nanoparticles (PPA NPs) onto the surface of MSCs via bioorthogonal chemistry (Fig. 6A). This design leveraged the homing effect of MSCs to achieve joint targeting and triggered pH-responsive H_2_ release within the acidic lesion microenvironment (Fig. 6B). It effectively scavenged ROS, promoted macrophage polarization toward the M2 phenotype, and facilitated cartilage regeneration in a rat OA model (Fig. 6C) [43]. Wang et al. developed a multifunctional injectable hydrogel (mPDA@Mel-AB OCS/CMC-BG), in which mesoporous polydopamine nanoparticles (mPDA) co-loaded with melatonin and AB were incorporated into an oxidized chondroitin sulfate (OCS) and carboxymethyl chitosan (CMC) hydrogel network through Schiff base reactions. In a rat OA model, treatment with this multifunctional hydrogel significantly reduced the OARSI score from approximately 14.5 (untreated OA control) to about 4.5, representing a ∼69% improvement in cartilage integrity. This result quantitatively confirms that the synergistic pH-responsive H_2_ release and melatonin-mediated anti-oxidative effects not only alleviated oxidative stress and inflammation but also led to substantial structural repair of the damaged articular cartilage [168]. In another study, researchers designed a multifunctional H_2_-releasing nanozyme (Se-HMPB@AB@COS) based on hollow mesoporous Prussian blue nanoparticles, in which H_2_ release from AB was triggered by the acidic lesion microenvironment to achieve targeted and sustained H_2_ delivery. In a mouse OA model induced by anterior cruciate ligament transection, this system effectively delayed cartilage degeneration and disease progression [169]. Furthermore, Zhang et al. proposed a nanoconfinement strategy, immobilizing AB molecules within oxygen-deficient mixed-phase titanate nanocrystals (HyPT) via a one-end anchored docking (OEAD) approach, restricting them to slow hydrolysis only upon interlayer water intrusion (Fig. 6D). This design enabled sustained H_2_ release for up to 11 days under high-glucose conditions simulating a diabetic environment and, in synergy with preloaded Mg^2+^, not only alleviated diabetes-associated oxidative stress but also significantly promoted innervation and vascularization (Fig. 6E), achieving enhanced bone regeneration in both diabetic and normoglycemic rabbit femoral defect models (Fig. 6F and G) [105]. In summary, these studies demonstrate that AB-based materials, through the modulation of H_2_ release kinetics and the integration of synergistic factors, provide effective therapeutic strategies for bone repair under complex pathological conditions.

A cross-comparison reveals that for the first three material types (Mg-based, Ca-based, and Si-based), the degradation products (Mg^2+^, Ca^2+^, and silicic acid) generated during H_2_-releasing hydrolysis possess well-known osteogenic activity. This enables a combination of H_2_-mediated therapy with ion-mediated osteogenesis. In contrast, AB itself lacks osteogenic function, and its degradation products (borate/boric acid) pose potential nephrotoxicity, significantly raising the challenges for material design and clinical translation. Notably, the effects of these H_2_-releasing reactions on the local microenvironmental pH are system-dependent. Mg- and Ca-based systems form metal hydroxides during hydrolysis, which may contribute to local alkalinization. In contrast, Si-based systems generate soluble silicon species during H_2_ release, with only limited overall pH changes reported under near-neutral aqueous conditions. AB hydrolysis likewise does not inherently involve direct OH^−^ generation, with its boron-containing products existing as pH-dependent boric acid/borate species.

### 5.2 Application of physically responsive H_2_-releasing biomaterials in bone regeneration

#### 5.2.1 Photocatalytic H_2_-releasing biomaterials

Although research on photocatalytic H_2_-releasing biomaterials for bone tissue engineering remains in its early stages, their ability to achieve light-triggered and controllable H_2_ release via exogenous light irradiation endows them with distinct application potential compared to spontaneous hydrolysis or acid-responsive systems. For example, Shi et al. developed a multifunctional artificial periosteum (Fg/PDA@g-C_3_N_4_) based on polydopamine-modified graphitic carbon nitride composites crosslinked with fibrin (Fg) hydrogel. Under NIR irradiation, PDA@g-C_3_N_4_ catalyzed the decomposition of water molecules to generate H_2_ and O_2_ in situ, thereby alleviating oxidative stress and hypoxia in the transplanted microenvironment. In an aged diabetic rat cranial defect model, Fg/PDA@g-C_3_N_4_ scaffolds loaded with aged human nasal mucosa-derived ectodermal stem cells markedly improved stem cell survival and retention rates, and further enhanced osteogenic differentiation and angiogenesis. Micro-CT analysis at 8 weeks post-implantation revealed a 4.1-fold increase in BV/TV relative to the control (28.6 ± 5.1% vs. 6.9 ± 1.9%) [42]. The above research demonstrates that photocatalytic H_2_-releasing systems offer a promising strategy for bone defect repair under complex pathological conditions by modulating the microenvironment.

#### 5.2.2 Piezoelectric-responsive H_2_-releasing biomaterials

**Fig. 7:**
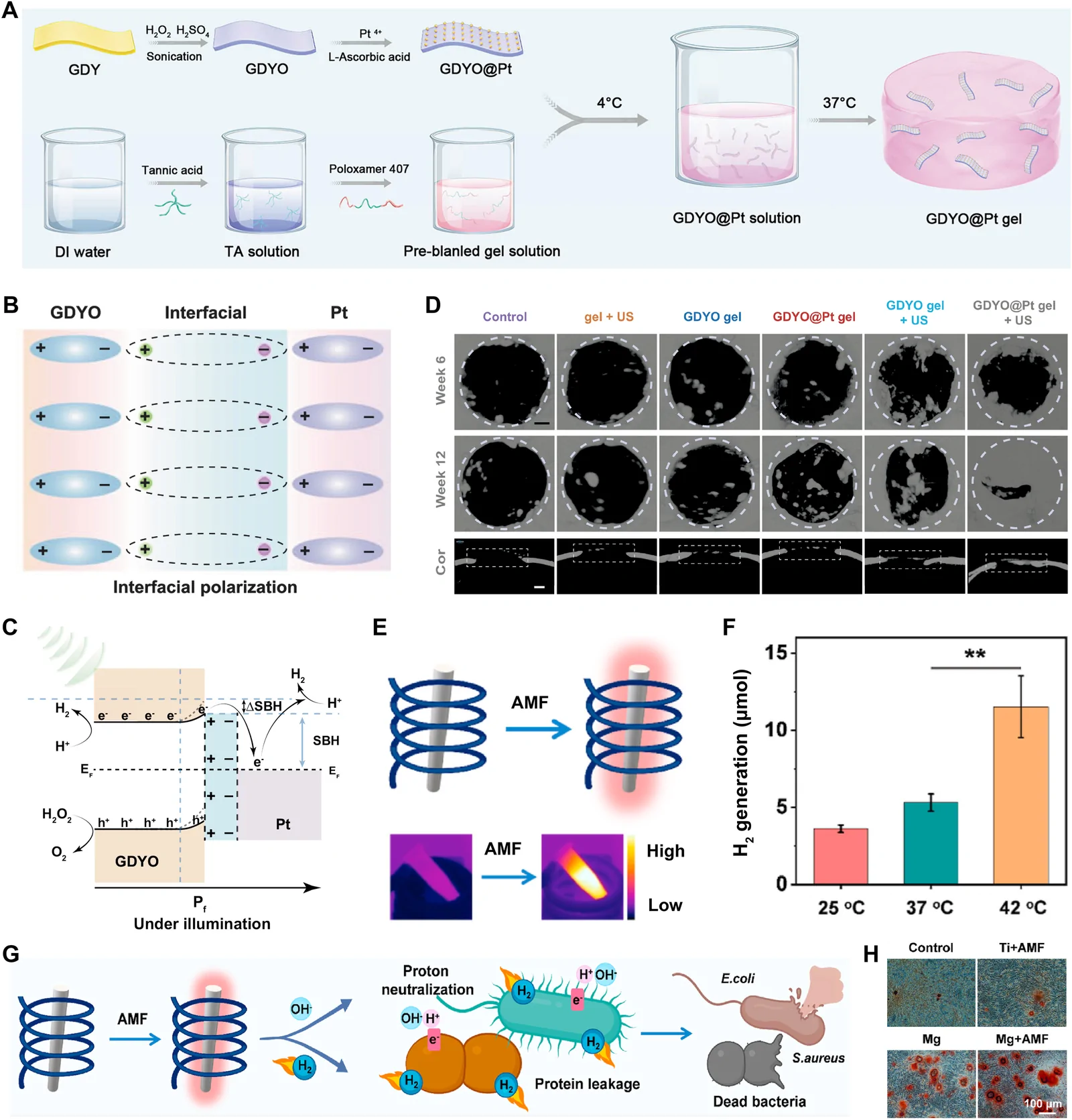
Representative studies of physically responsive H_2_-releasing biomaterials for bone regeneration. (A) Fabrication protocol of GDYO@Pt nanosheets and their hydrogel composite. (B) Schematic representation of the polarization mechanism of GDYO@Pt nanosheets. (C) Piezocatalytic mechanism in GDYO@Pt (SBH: Schottky barrier height). (D) Representative microscopic CT images of the cranial defect site at 6 and 12 weeks after implantation in different treatment groups. Scale bars: 0.5 mm. White circles represent defect boundaries. Reproduced with permission [40]. Copyright 2025, The authors. (E) Schematic diagram showing the eddy-thermal effect of Mg implants under an alternating magnetic field (AMF). (F) H_2_ generation performance of Mg rods in PBS at different temperatures. (G) The antibacterial mechanism of Mg rods under an AMF via H_2_ and proton depletion. (H) Alizarin red staining of MC3T3-E1 cells after 14 days of osteogenic differentiation. Reproduced with permission [39]. Copyright 2024, The authors.

Piezoelectric materials can promote bone regeneration by converting mechanical stimuli into electrical signals [174]. Building on this, piezoelectric catalytic materials further integrate H_2_ generation functionality to synergistically regulate the pathological microenvironment. For example, Song et al. constructed a platinum-decorated graphdiyne oxide (GDYO@Pt) Schottky junction and encapsulated it within a thermosensitive hydrogel (Fig. 7A). They leveraged the built-in electric field induced by interfacial dipoles to break material symmetry, significantly enhancing the piezoelectric effect (PE) (Fig. 7B). Under ultrasound stimulation, polarized charges at the Schottky junction drove electron transfer from GDYO to Pt, promoting H_2_ production (Fig. 7C), while the corresponding holes and nanozyme activity catalyzed H_2_O_2_ decomposition into O_2_, synergistically alleviating oxidative stress. In a mouse calvarial defect model, the ultrasound-activated GDYO@Pt gel significantly promoted bone regeneration (Fig. 7D), achieving a new bone area ratio of 75.37%, a BV/TV of 69.00%, and a BMD of 0.658 g/cm^3^ at 12 weeks [40]. Recently, Wang et al. constructed Piezo-MnO_2_ motors by loading barium titanate nanoparticles (BTO NPs) into mesoporous silica nanoparticles (MSN@BTO) and coating them with asymmetric MnO_2_ shells. The unique Janus structure endowed the motors with active motion capability, enabling them to utilize the elevated H_2_O_2_ in the OA microenvironment as fuel for self-propulsion, thereby achieving enhanced penetration into the dense cartilage matrix. Upon ultrasonic stimulation, the piezoelectric BTO NPs generated electrical signals via the piezoelectric effect, driving water splitting to produce H_2_, while MnO_2_ catalyzed the decomposition of excess H_2_O_2_ into O_2_ in the OA microenvironment, synergistically mitigating oxidative stress. In a monoiodoacetate (MIA)-induced rat OA model, this system significantly promoted bone repair and improved cartilage integrity [160]. Such piezoelectric responsive H_2_-releasing materials preliminarily achieve stimulus-responsive H_2_ release and multifunctional microenvironment modulation. By integrating energy conversion with biological regulation, they provide a new strategy for developing intelligent bone repair materials.

#### 5.2.3 Magnetothermal-responsive H_2_-releasing biomaterials

Magnetothermal-responsive H_2_-releasing systems exhibit distinct advantages in the field of intelligently controlled H_2_ delivery, owing to their non-contact energy transfer, deep tissue penetration, and spatiotemporally controllable external field manipulation. Compared with H_2_ release methods triggered by the endogenous microenvironment, this system achieves a paradigm shift in H_2_ release kinetics from passively responding to environmental fluctuations to active, controlled regulation. Although research on such materials in bone tissue engineering remains relatively limited, their potential advantages have begun to emerge. A representative example is the innovative design proposed by Yang et al., which utilizes the eddy-thermal effect driven by an AMF to induce localized heating, thereby accelerating the corrosion reaction kinetics of a Mg rod with the surrounding bodily fluid (Fig. 7E and F). This enables controlled H_2_ release along with the simultaneous generation of OH^−^ and Mg^2+^. In a mouse osteomyelitis model, this system employed H_2_ to scavenge excessive ROS and attenuate local inflammation, synergized with OH^−^ to enhance antibacterial efficacy, and leveraged the osteogenic activity of Mg^2+^ to achieve integrated therapy combining infection control (Fig. 7G) and bone regeneration (Fig. 7H) [39].

Physically responsive materials provide important strategies for bone repair. Photocatalytic and piezoelectric materials, leveraging their unique capacity for co-delivering H_2_ and O_2_, exhibit synergistic advantages in alleviating hypoxia and neutralizing toxic ROS in bone defect areas. Moreover, light or ultrasound can serve as external switches to achieve active control of H_2_ release. However, both material types face the physical limitation of energy attenuation that increases exponentially with tissue depth, making their efficacy unclear in deep bone defects. In contrast, magnetothermal-responsive materials activated by AMF exhibit superior tissue penetration with less energy attenuation. Nonetheless, they still face challenges such as insufficient heat generation due to the poor thermal conversion capability of existing materials [175].

In addition to the aforementioned chemically and physically responsive H_2_-releasing biomaterials, some studies have focused on passive H_2_ release strategies that do not require conditional stimuli but instead rely on inherent diffusion. For instance, Liu et al. developed a bone-targeted nanocomposite, A-Z@Pd(H), in which reducible H_2_ was loaded onto palladium nanocubes, while bone targeting was achieved through ZIF-8 encapsulation and alendronate (Ald) modification. H_2_ release from this system primarily depends on physical desorption and diffusion, exhibiting a time-dependent sustained release profile. In an OVX-induced mouse model of osteoporosis, this material effectively improved the bone microenvironment, increased bone mineral density, and suppressed osteoclast activity [166].

In summary, existing studies on H_2_-releasing biomaterials indicate that the key role of H_2_ in bone repair lies in reshaping the pathological microenvironment through mechanisms such as redox regulation and anti-inflammatory effects. This creates favorable conditions for other active components, such as metal ions, to exert efficient osteogenic functions. Depending on the pathological context, material design has evolved toward context-specific strategies. In infection models, the focus is on rapid H_2_ production and synergistic antibacterial effects [39,164]. In aging and diabetic models, the emphasis shifts to long-term sustained release and the reconstruction of vascular, neural, and stem cell microenvironments [38,103]. In osteoporosis models, combined regulation of H_2_ and anti-resorptive agents is being explored [37]. These distinct therapeutic demands have motivated the development of both chemically responsive and physically triggered H_2_-releasing systems. Chemically responsive systems generally enable autonomous H_2_ generation with relatively simple designs, but may suffer from local alkalization and limited control over release profiles. In contrast, physically responsive systems offer greater flexibility for controlled regulation of H_2_ release, but at the cost of increased engineering complexity and dependence on external stimuli. Despite these advances in material design, the H_2_-specific contribution has not been consistently evaluated across published studies. Accordingly, several entries in Table 1 are marked as “NA”, primarily because the original studies lacked appropriate composition-matched non-H_2_ controls. Beyond these limitations in evaluating H_2_-specific effects, both categories continue to face common challenges in degradation behavior, long-term biosafety, manufacturing reproducibility, and translational feasibility. Overall, this field remains at an early stage of development, and studies targeting specific bone defect diseases are still relatively limited.

## 6 Cross-disciplinary insights: translating H_2_-releasing biomaterials to bone regeneration

Current research on H_2_-releasing biomaterials is developing rapidly, with an increasing number of disease models being explored and a growing emphasis on multi-mechanism synergistic approaches. The successful application of H_2_-releasing biomaterials in fields such as oncology, wound repair, neurodegenerative diseases, and metabolic disorders has not only validated the broad potential of H_2_ therapy but have also spurred many innovative material-design strategies. These cross-disciplinary practices provide important references for bone tissue engineering (Table 2).

**Table 2:** Cross-disciplinary design paradigms of smart H_2_-releasing biomaterials for bone regeneration.

Material types	Cross-disciplinary strategy	Potential for bone regeneration	Refs
Chemically responsive	Inspired by Fe-based TME-activated systems for acid-triggered, selective in situ H_2_ generation with coupled iron ion/redox regulation	Pathological acidic–inflammatory niches in bone defects may enable selective activation of Fe-driven H_2_ release, integrating gas therapy, ionic bioactivity, and microenvironment remodeling for multimodal repair	[110,143]
Photocatalytic	UCNPs convert deeply penetrating NIR (980 nm) into visible light for remote activation; simultaneous consumption of local metabolic substrates generates H_2_	In situ H_2_ release in deep bone defects; dual function for “metabolic modulation-gas therapy” in hyperglycemic microenvironments	[126,133,134]
Ultrasound-responsive	Sonodynamic H_2_ generation	Combined with clinical LIPUS to provide dual regulation of physical stimulation and local H_2_ release	[125]

Abbreviations: TME: tumor microenvironment; UCNPs: upconversion nanoparticles; NIR: Near Infrared; LIPUS: low-intensity pulsed ultrasound.

### 6.1 Chemically responsive H_2_-releasing biomaterials

**Fig. 8:**
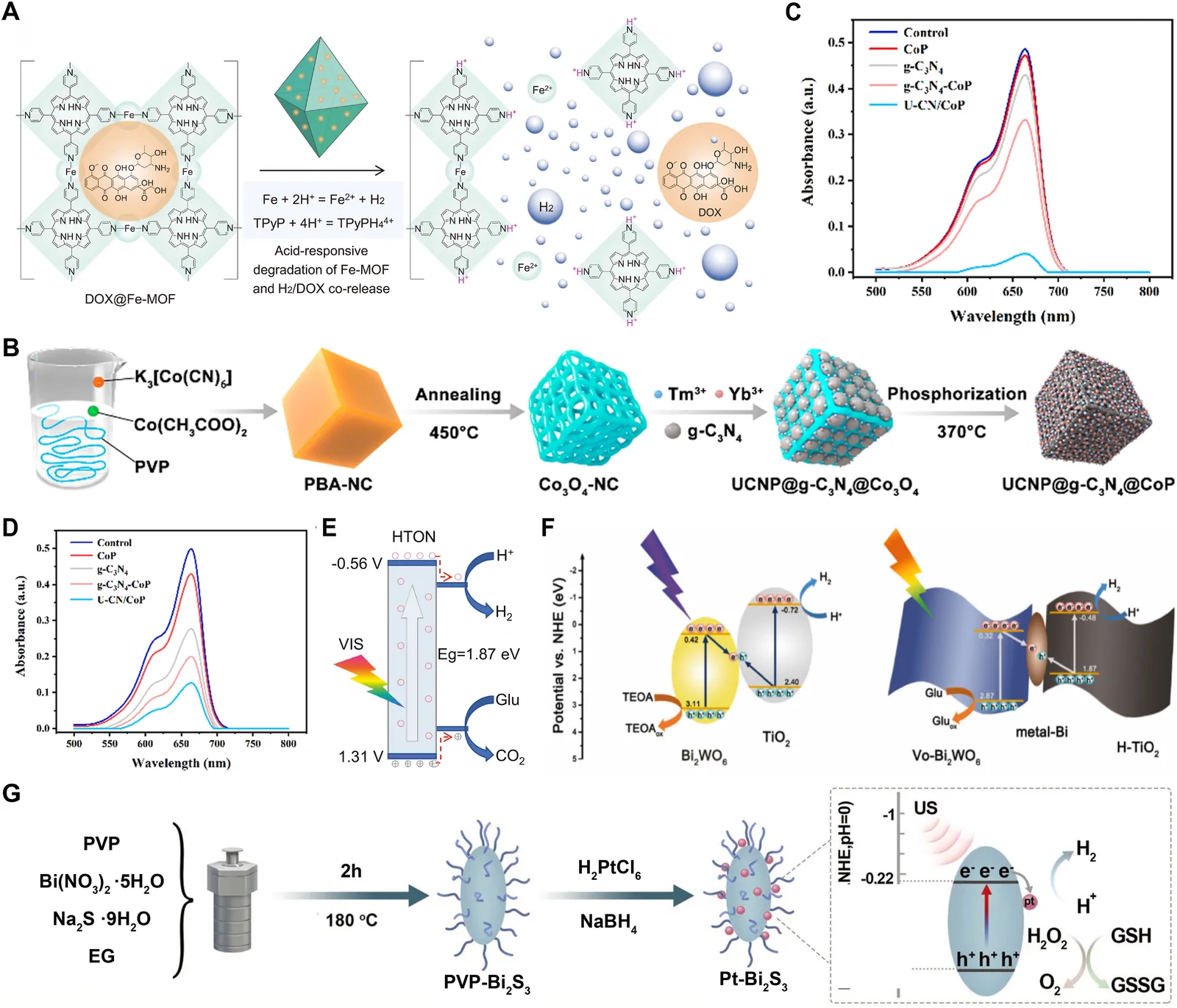
Inspirational design paradigms of H_2_-releasing systems for bone repair: Lessons from other biomedical fields. (A) Schematic illustration of acid-controlled H_2_/drug release from DOX@Fe-MOF. Reproduced with permission [143]. Copyright 2022, The Authors. (B) Diagrammatic representation of the U-CN/CoP synthesis pathway. (C)Absorbance of MB solution containing CoP, g-C_3_N_4_, g-C_3_N_4_-CoP, and U-CN/CoP nanoparticles at 664 nm under 450 nm laser radiation. (D) Absorption of an MB solution containing CoP, g-C_3_N_4_, g-C_3_N_4_-CoP, and U-CN/CoP nanoparticles at 664 nm under 980 nm laser radiation. Reproduced with permission [126]. Copyright 2023, American Chemical Society (ACS). (E) Band structures (versus normal H_2_ electrode (NHE)) of HTON and the schematic illustration of the mechanism for VIS-photocatalytic H_2_ generation and glucose consumption. Reproduced with permission [133]. Copyright 2022, The authors. (F) Band structure of Bi_2_WO_6_/TiO_2_ and Bi/Bi_2_WO_6_/H-TiO_2_. Reproduced with permission [134]. Copyright 2025, Elsevier. (G) Schematic illustration of the mechanism of sonocatalytic H_2_ evolution from Pt-Bi_2_S_3_ nanocatalysts. Reproduced with permission [125]. Copyright 2023, Wiley-VCH GmbH.

In bone tissue engineering, existing chemically responsive H_2_-releasing materials (mainly based on Mg, Ca, or Si) generate H_2_ primarily through hydrolysis in body fluids, which lacks selectivity for the inflammatory and acidified microenvironment at bone defect sites. In contrast, Fe-based materials release H_2_ predominantly under acidic conditions and show minimal activity under normal physiological settings. For example, in tumor therapy, Fe-based H_2_-releasing systems, such as Fe@CMC nanoparticles [110] and Fe-MOF nanocrystals (Fig. 8A) [143], have already been applied for in situ H_2_ generation in acidic tumor microenvironment. This acid-triggered feature is particularly relevant to bone defects, where localized acidosis frequently accompanies inflammation. Moreover, Fe-based materials without H_2_-releasing functions already have a well-established foundation for application in bone tissue engineering and exhibit favorable pro-osteogenic activity [[176], [177], [178]]. Therefore, acid-responsive Fe-based H_2_-releasing systems could enable preferential activation of H_2_ release within inflamed or infected regions, offering a potential strategy for microenvironment-selective H_2_ release in bone regeneration.

### 6.2 Physically responsive H_2_-releasing biomaterials

Photocatalytic materials currently applied in bone tissue engineering, such as g-C_3_N_4_, have shown potential in bone repair through polydopamine modification to broaden their light response range [42]. However, their catalytic activity still primarily relies on electron excitation from the valence band to the conduction band. When applied to bone defects located in deep anatomical sites (e.g., those covered by substantial soft tissue), these systems face a critical limitation: although NIR photons offer superior tissue penetration, their relatively low energy prevents effective excitation of valence band electrons across the semiconductor bandgap. This mismatch between photon energy and the material's optical bandgap severely limits H_2_ production efficiency in deep tissues. The introduction of UCNPs offers a promising technological pathway to overcome this energy barrier. For instance, Ge et al. developed a UCNP@CoP@g-C_3_N_4_ composite system in which UCNPs efficiently convert deeply penetrative NIR light (980 nm) into visible light capable of exciting g-C_3_N_4_ (Fig. 8B–D), thereby enabling remotely driven in situ water splitting and H_2_ generation in deep brain tissue [126]. Such a strategy is highly relevant to bone repair, as it provides a feasible pathway to achieve non-invasive, deep-tissue activation of H_2_-releasing systems, thereby overcoming a major translational barrier in treating deep bone defects.

Current material strategies for diabetic bone defect repair mostly focus on intervening in downstream pathological phenotypes, such as alleviating oxidative stress and inhibiting excessive inflammation, but fail to effectively address hyperglycemia, the upstream core driver of microenvironmental imbalance. It is noteworthy that emerging photocatalytic H_2_-releasing materials have shown unique potential to directly regulate the pathological microenvironment at the metabolic level. For example, Chen et al. and Shen et al. designed photocatalytic systems that utilize locally excessive glucose as a substrate, consuming glucose through a catalytic reaction while simultaneously generating H_2_ (Fig. 8E and F), where H_2_ exerts anti-inflammatory and anti-oxidative effects, while glucose consumption contributes to modulation of the hyperglycemic microenvironment, thereby yielding a synergistic therapeutic effect in diabetic wound healing [133,134]. This strategy directly targets the upstream hyperglycemia rather than downstream consequences, which is highly desirable for treating bone healing disorders associated with metabolic diseases.

In current research on H_2_-releasing materials for bone repair, ultrasound is mainly used as a mechanical stress source to activate piezoelectric materials. However, in other disease models, ultrasound has been utilized to catalyze H_2_ production. For instance, Yuan et al. constructed a Pt-Bi_2_S_3_ heterojunction by uniformly depositing platinum nanoparticles on Bi_2_S_3_ nanorods, which catalyzes water splitting under ultrasound to achieve continuous H_2_ generation (Fig. 8G), synergistically enhancing antitumor effects through combined sonocatalytic and H_2_ therapy [125]. Furthermore, low-intensity pulsed ultrasound (LIPUS) is already widely used as an adjunctive treatment for accelerating bone fracture healing through mechanical stimulation and cellular signaling regulation [179]. These research advances provide important insights for developing ultrasound-responsive H_2_-releasing materials: combining catalytic H_2_ release functions with ultrasound therapy holds the potential to further amplify therapeutic effects. For example, developing ultrasound-triggerable H_2_-releasing bone implant materials could enable LIPUS treatment to simultaneously activate localized H_2_ release while providing mechanical stimulation, thereby synergistically promoting bone regeneration.

## 7 Challenges and future directions of H_2_-releasing biomaterials for bone regeneration

Despite the promising therapeutic potential of H_2_-releasing biomaterials in bone regeneration, several critical challenges continue to impede deeper mechanistic understanding and clinical translation. The major limitations include the lack of quantitative H_2_ evaluation systems and reliable in vivo monitoring strategies, insufficient understanding of the balance between therapeutic efficacy and safety during H_2_ generation, and difficulties in achieving scalable manufacturing consistency and long-term biosafety.

**Fig. 9:**
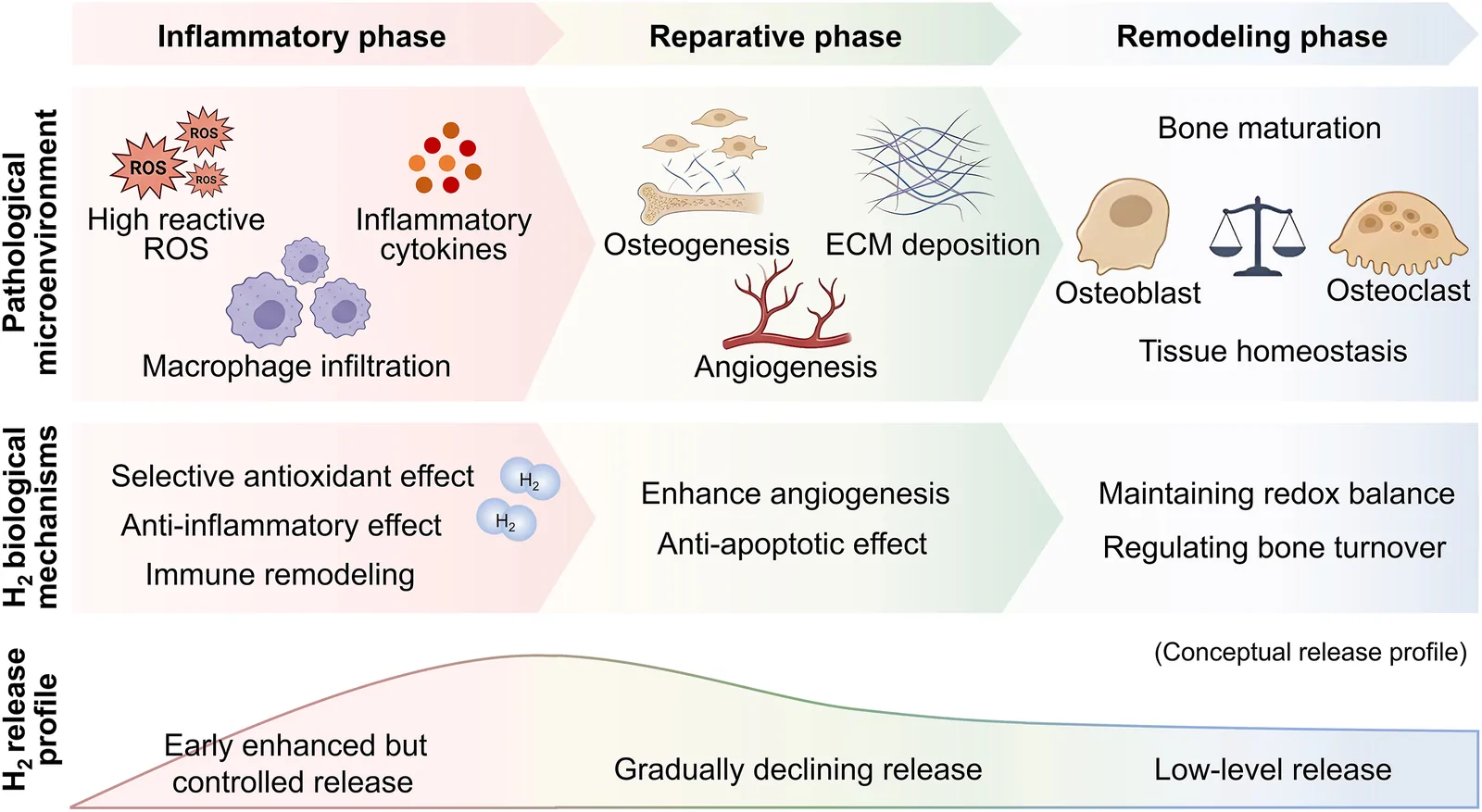
Conceptual framework of stage-adaptive H_2_ release during bone regeneration. The release profile is schematically illustrated to represent a proposed stage-matched design strategy rather than an experimentally validated release pattern. Created with BioRender.com.

First, although localized, controllable, and sustained H_2_ release has been a central research focus of H_2_-releasing biomaterials for bone regeneration, the development of stage-adaptive H_2_ release strategies remains a significant challenge. The current literature predominantly offers qualitative descriptors such as “controlled” or “sustained release” without defining biologically relevant concentration windows. Only a limited number of studies have reported quantitative metrics related to H_2_ generation or release behavior in bone-regenerative applications. For instance, using an in vitro gas collection method, Chen et al. reported that CaSi_2_ nanoparticles exhibited a H_2_ production capacity of 911 mL/g upon physiological hydrolysis [38], while Pang et al. reported a volume concentration of 8.6% for injectable short fibers based on gas yield from 100 mg samples in PBS (pH 7.4) [34]. However, the lack of unified measurement standards, including differences in detection units and conditions, makes cross-study comparisons difficult. More importantly, the biological requirements for H_2_ may vary throughout the inflammatory, repair, and remodeling phases of bone healing. Yet, the quantitative criteria required to guide controlled H_2_ delivery remain poorly defined, particularly with respect to effective concentration windows, dose-response relationships, and stage-specific release kinetics. These limitations hinder the establishment of a quantitative framework capable of matching H_2_ release profiles with physiological demands. We therefore propose a conceptual stage-adaptive framework (Fig. 9) to correlate the dynamic biological requirements of bone healing with idealized H_2_ release profiles.

Second, a fundamental reason for the lack of quantitative H_2_ metrics is that multiple physiological barriers profoundly alter H_2_ transport and clearance in the body. Consequently, in vitro release kinetics cannot directly predict in vivo efficacy. On one hand, biological processes after material implantation, such as protein adsorption [180,181] and immune cell phagocytosis and encapsulation [182,183], dynamically remodel the material interface. These processes alter material degradation pathways and interfacial reaction behaviors, thereby affecting the actual H_2_ generation rate and its spatial distribution. On the other hand, existing detection methods (e.g., gas chromatography, methylene blue probes, and in situ microelectrodes) are limited by diffusion constraints, signal interference, and insufficient spatial resolution in three-dimensional porous scaffold systems [[184], [185], [186], [187]]. Several imaging and biosensing strategies have enabled preliminary in vivo monitoring of gaseous molecules [188]. However, these approaches have been primarily developed for gasotransmitters such as NO, H_2_S, and CO rather than H_2_. A technical platform capable of in situ, real-time, and quantitative detection of H_2_ in deep living tissues remains immature [189,190]. This limitation further hinders the establishment of reliable links between H_2_ release kinetics and stage-dependent biological responses. Accordingly, future research should prioritize developing in vitro evaluation systems that better simulate the in vivo microenvironment. When combined with in vivo imaging and sensing technologies, such systems could help clarify the spatiotemporal distribution of H_2_, define effective concentration thresholds, and determine stage-specific release kinetics within the bone repair microenvironment. These insights would, in turn, inform the rational design of adaptive H_2_-releasing biomaterials.

Third, chemically and physically responsive H_2_-releasing systems face distinct limiting factors. For chemically responsive materials, particularly Mg- and Ca-based systems, hydrolysis inevitably generates OH^−^ and H_2_. This raises the primary safety issues, including local alkalization and uncontrolled gas release. Rapid OH^−^ accumulation elevates local pH and may impair cellular function [191,192]. Moreover, excessive H_2_ evolution can exceed tissue clearance, producing gas cavities that delay healing and potentially cause microvascular gas embolism [[193], [194], [195]]. Current strategies primarily regulate release kinetics through materials engineering approaches, such as the construction of hydrogel buffering systems and nanoconfinement designs, to achieve sustained release and mitigate potential side effects associated with rapid reactions [36,105]. However, unified physiological safety thresholds for OH^−^ release rate or H_2_ generation rate are still lacking, and most evaluations still rely on indirect indicators such as local pH changes, bubble formation, and histological outcomes. Notably, there is currently no evidence that H_2_ itself is directly cytotoxic. The risks primarily stem from burst gas release and local alkalization, rather than from H_2_ itself. Future efforts should establish a quantitative correlation between release kinetics and biological effects to better guide the design of material degradation and gas generation profiles. By contrast, physically responsive materials rely on external energy input to achieve stimulus-responsive H_2_ release and carry a lower burst-release risk, but are limited by inadequate energy delivery. Most existing studies have been conducted in small animals with limited tissue thickness, where light or ultrasound attenuation is minor and related challenges have not yet fully emerged. As research advances toward large-animal models and clinical translation, the complex anatomy, thick soft tissues, and dense bone barriers will markedly increase energy attenuation and scattering, reducing the efficiency of energy delivery. Future efforts should therefore focus on enhancing tissue penetration and local energy utilization.

Fourth, from a clinical translation perspective, balancing design complexity with manufacturing and regulatory requirements remains a challenge for H_2_-releasing biomaterials. Chemically responsive systems rely on pH- or hydrolysis-triggered reactions to generate H_2_. During chemistry, manufacturing, and controls (CMC) scale-up, this responsive nature may increase sensitivity to material composition and processing conditions, posing challenges for scalability, batch-to-batch reproducibility, and the establishment of good manufacturing practice (GMP)-compliant manufacturing processes [[196], [197], [198]]. Moreover, these materials may undergo structural alterations and performance loss under conventional sterilization conditions [199,200], making sterilization stability an important consideration for product development and commercialization. Physically responsive systems are designed for stimulus-responsive H_2_ release with the potential for spatiotemporal regulation but rely heavily on external devices. Ensuring their energy delivery efficiency and dose reproducibility across heterogeneous tissue environments remains difficult, which in turn increases clinical complexity. Furthermore, the regulatory classification of H_2_-releasing biomaterials may be challenging. Products that integrate biomaterial matrices, H_2_-generating components, and external activation devices could be regulated as combination products, which would entail more complex approval pathways [201]. Beyond these manufacturing and regulatory considerations, evidence supporting long-term safety remains limited. While many studies have reported no apparent systemic toxicity via H&E staining of major organs post-implantation, these assessments are mostly short-term (3-12 weeks) in small animals (mouse, rat, and rabbit). Systematic data on long-term in vivo metabolism, biodistribution, and chronic immunotoxicity are still lacking, impeding clinical translation. Future work should therefore focus on achieving controllable H_2_ release while simultaneously improving manufacturing consistency, sterilization compatibility, and long-term biosafety to facilitate clinical translation.

## 8 Conclusions

Bone defect repair under pathological conditions remains a major challenge. This regenerative process is governed not by a single factor but by the dynamic interplay among oxidative stress, inflammatory responses, vascular dysfunction, and impaired osteogenic signaling. In this context, H_2_-releasing biomaterials offer a distinctive strategy for bone regeneration by integrating redox regulation with multifunctional microenvironmental modulation. Unlike conventional anti-oxidative systems that broadly suppress ROS activity, H_2_ may preferentially attenuate highly reactive and cytotoxic species, while minimally interfering with physiologically important redox signaling. Together with its anti-inflammatory, pro-angiogenic, anti-apoptotic, and osteoclast-regulating effects, H_2_ shows considerable potential as a fundamental regulator of the pathological bone repair microenvironment.

This review systematically summarizes the pathological characteristics of bone defects and discusses the biological basis of H_2_-mediated intervention in bone regeneration. From the perspective of material engineering, current design strategies for H_2_-releasing biomaterials are classified into chemically responsive and physically responsive systems. Recent advances indicate that H_2_-releasing systems are gradually evolving from simple gas-generation platforms toward more sophisticated biomaterials capable of localized, sustained, and responsive therapeutic regulation. In particular, the combination of H_2_ delivery with bioactive ions, osteogenic agents, scaffolds, and external physical stimulation has expanded the functional versatility of these materials. This integration provides new opportunities for treating complex bone defects under inflammatory, infectious, diabetic, or osteoporotic conditions. Despite these encouraging advances, the field remains at an early stage of development. Current studies have demonstrated the feasibility of H_2_-based microenvironmental regulation. However, translating these systems into clinically reliable therapies still requires deeper mechanistic understanding, improved quantitative evaluation of H_2_ release behavior, and enhanced integration between material design and the dynamic biological requirements of bone healing. Future development should therefore move beyond empirically driven gas-release systems toward mechanism-guided biomaterials with improved controllability, adaptability, and translational compatibility.

Overall, H_2_-releasing biomaterials provide a microenvironment-oriented therapeutic strategy and represent a promising direction for bone tissue engineering. Nevertheless, current evidence remains largely preclinical, with most studies conducted in small-animal models. Further studies are needed to better distinguish H_2_-specific effects from other material-derived functions, establish standardized approaches for evaluating H_2_ dosage and release behavior, and clarify long-term biodegradation and biosafety profiles. With continued advances in mechanistic understanding, responsive material design, and translational engineering, H_2_-based biomaterials are expected to contribute to next-generation strategies for bone regeneration.

## CRediT authorship contribution statement

**Yanshan Chen:** Writing – review & editing, Writing – original draft, Visualization, Data curation. **Yuanyuan Yin:** Writing – review & editing, Writing – original draft, Supervision, Funding acquisition. **Baoyi Huang:** Data curation. **Jingqiu Chen:** Data curation. **Tingting Shu:** Data curation. **Xiaohui Xu:** Writing – review & editing, Supervision, Funding acquisition. **Guangyue Li:** Writing – review & editing, Supervision, Funding acquisition.

## Ethics approval and consent to participate

This review article does not require any ethical approval or informed consent for publication.

## Declaration of competing interest

The authors declare no competing nor financial interests, or personal relationship that influenced this paper.
